# Supplementary material for: Human OPRM1 and murine Oprm1 promoter driven viral constructs for genetic access to μ-opioidergic cell types
Source: Nat Commun. 2023 Sep 13;14:5632. doi: 10.1038/s41467-023-41407-2 (PMC10499891; doi:10.1038/s41467-023-41407-2)
Supplement: Supplementary file 1 — Supplementary Information [file 41467_2023_41407_MOESM1_ESM.pdf]

nature communications

Supplemental Information

Human *OPRM1* and murine *Oprm1* promoter driven viral constructs for genetic access to μ-opioidergic cell-types

Gregory J. Salimando[1,2], Sébastien Tremblay[1,2], Blake A. Kimmey[1,2], Jia Li[3], Sophie Rogers[1,2], Jessica A. Wojick[1,2], Nora M. McCall[1,2], Lisa M. Wooldridge[1,2], Amrith Rodrigues[4], Tito Borner[1,5], Kristin L. Gardiner[6], Selwyn S. Jayakar[7], Ilyas Singeç[8], Clifford J. Woolf[7], Matthew R. Hayes[1,5], Bart C. De Jonghe[1,5], F. Christopher Bennett[1,9], Mariko L. Bennett[9], Julie A. Blendy[10], Michael L. Platt[1,2], Kate Townsend Creasy[4,5], William R. Renthal[3] , Charu Ramakrishnan[11], Karl Deisseroth[11,12,13,14*], Gregory Corder[1,2,*]

[1]Dept. of Psychiatry, Perelman School of Medicine, University of Pennsylvania, Philadelphia, PA, USA
[2]Dept. of Neuroscience, Mahoney Institute for Neurosciences, Perelman School of Medicine, University of Pennsylvania, Philadelphia, PA, USA
[3]Dept. of Neurology, Brigham and Women's Hospital and Harvard Medical School, Boston, MA, USA
[4]Translational Medicine and Human Genetics, Perelman School of Medicine, University of Pennsylvania, Philadelphia, PA, USA
[5]Dept. of Biobehavioral Health Sciences, School of Nursing, University of Pennsylvania, Philadelphia, PA, USA
[6]Dept. of Pathobiology, School of Veterinary Medicine, University of Pennsylvania, Philadelphia, PA, USA
[7]F.M. Kirby Neurobiology Center, Boston Children's Hospital and Harvard Medical School, Boston, MA, USA
[8]Stem Cell Translational Laboratory, National Center for Advancing Translational Sciences, National Institutes of Health, Rockville, MD, USA
[9]Division of Neurology, Dept. of Pediatrics, Children's Hospital of Philadelphia, Philadelphia, PA, USA
[10]Dept. of Systems Pharmacology & Translational Therapeutics, Perelman School of Medicine, University of Pennsylvania, Philadelphia, PA, USA
[11]CNC Program, Stanford University, Stanford, CA, USA
[12]Dept. of Bioengineering, Stanford University, Stanford, CA, USA
[13]Howard Hughes Medical Institute, Stanford University, Stanford, CA, USA
[14]Dept. of Psychiatry & Behavioral Sciences, Stanford University, Stanford, CA, USA

*Correspondence: gcorder@upenn.edu (G. C.), deissero@stanford.edu (K.D.)

Supplemental Figures: 1 – 16

a PROMO putative transcription factor binding sites - *Oprm1*

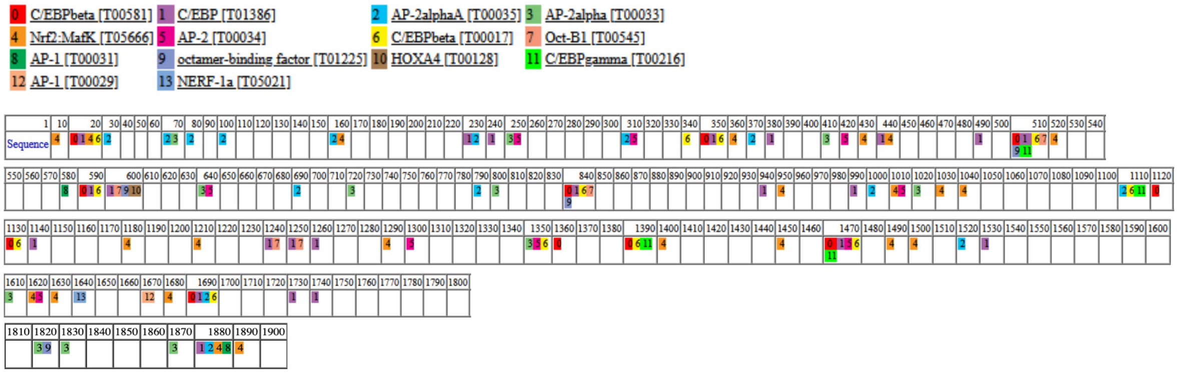

b Eukaroytic Promoter Database (EPD) & UCSC Genome Browser on Mouse - *Oprm1*

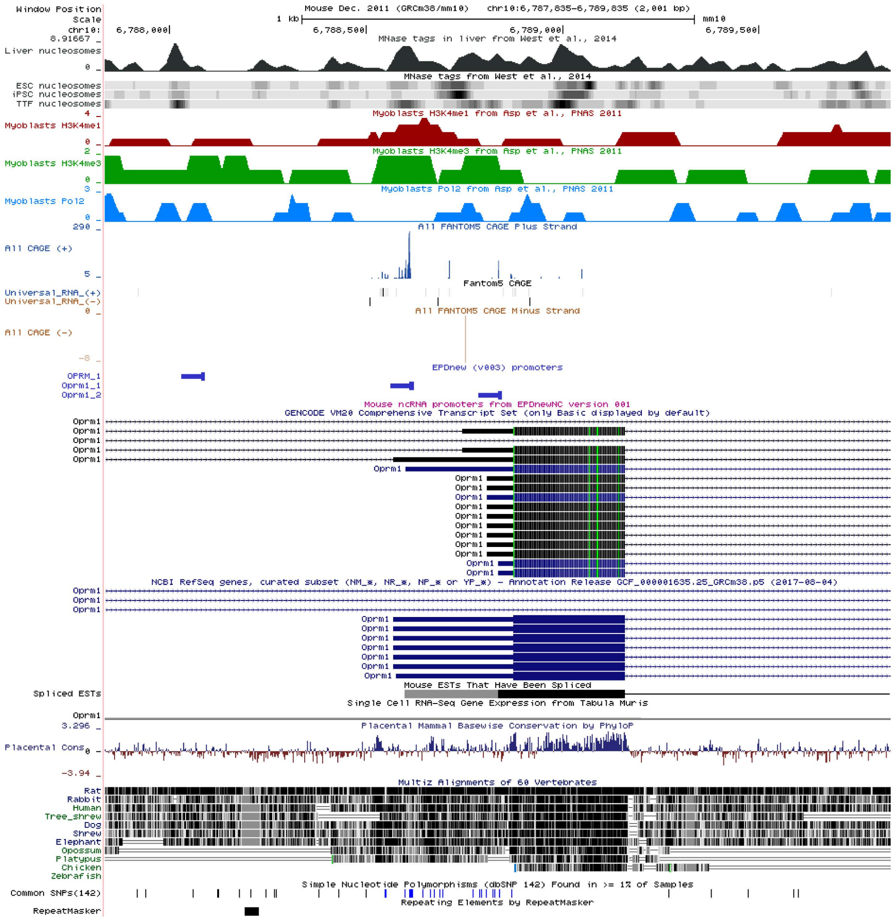

c *mMORp1*-eYFP construst design

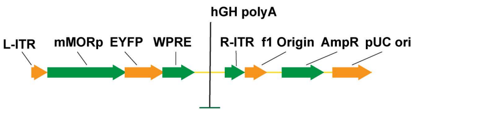

pAAV-*mMORp1*-EYFP-WPRE (6331 bp)

d *hMORp1*-eYFP construst design

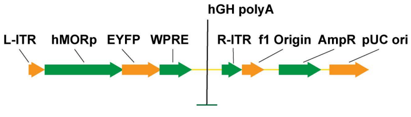

pAAV-*hMORp1*-EYFP-WPRE (6378 bp)

e Sequence homology of *mMORp*1 & *hMORp*1 constructs w/ native MORp sequences

	***mMORp*1 (Mouse)**		***hMORp*1 (Human)**	
**Target Genome**	Coverage Area	Percent Homology	Coverage Area	Percent Homology
Mouse	100%	100%	29%	66%
Rat	65%	84%	29%	67%
Human	21%	66%	100%	100%
Macaque	N/A	N/A	11%	96%

Supp. Fig.1 | Analysis of *Oprm1* genetic sequence with PROMO and EPD for identifying target regions for *mMORp1-4* construct designs and sequence homology. a, Readout run on the DNA sequence of the mouse *Oprm1* gene in order to identify putative transcription factor binding sites (TFBS) using the PROMO database[17] (http://alggen.lsi.upc.es/cgi-bin/promo_v3/promo/promoinit.cgi?dirDB=TF_8.3). Identified transcription factors are numbered and listed in a color-coded manner, while their putative location(s) within the *Oprm1* genetic sequence are tabulated below, including their position along the gene. **b,** Screen capture of readout produced by the Eukaryotic Promoter Database[18] (https://epd.epfl.ch//index.php) following a query for information on the promoter sequence of the *Oprm1* gene found in mice (*Mus musculus*). Pertinent information on the complete promoter sequence is provided and was used to guide the design of our *mMORp1-4* and *hMORp1* constructs to make them as complementary to desirable locations within the *Oprm1* and *OPRM1* promoter sequences, while avoiding regions containing potential splice sites that could hamper construct specificity. **c-d,** Linear plasmid maps for the *mMORp1-eYFP* (**c**) and *hMORp1-eYFP* (**d**) constructs. **e,** Tabulated results showing the percent total coverage and sequence homology for *mMORp*1 and *hMORp*1 when compared to the native *OPRM1*/*Oprm1* promoter sequence found in human, macaque, rat, and mouse. Sequence homology comparisons were conducted via the Basic Local Alignment Search Tool (BLAST) hosted by the NCBI (https://blast.ncbi.nlm.nih.gov/Blast.cgi).

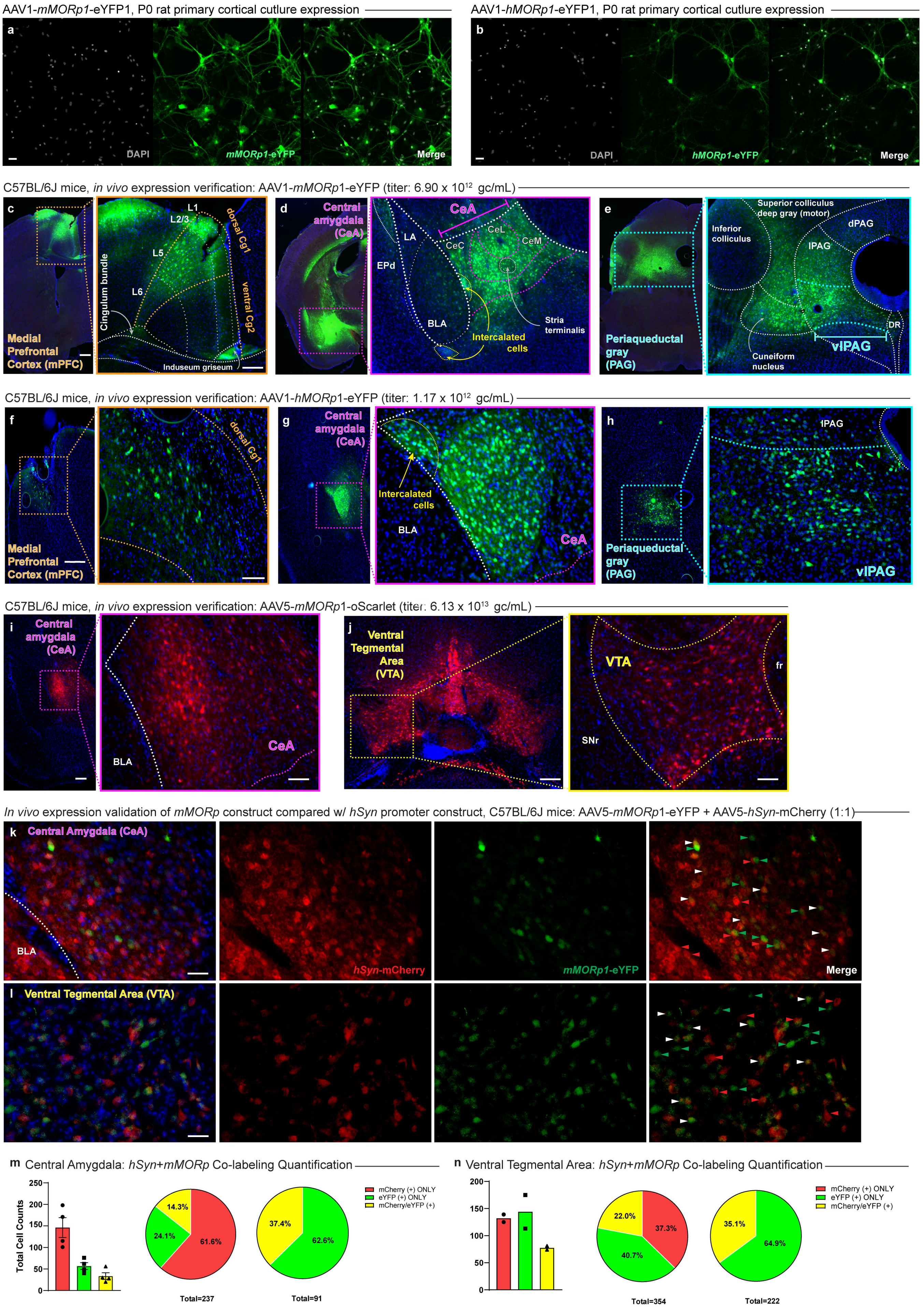
AAV1-mMORp1-eYFP1, P0 rat primary cortical cutlure expression
a
DAPI
mMORp1-eYFP
Merge
AAV1-hMORp1-eYFP1, P0 rat primary cortical cutlure expression
b
DAPI
hMORp1-eYFP
Merge
C57BL/6J mice, in vivo expression verification: AAV1-mMORp1-eYFP (titer: 6.90 x 10^12 gc/mL)
c
L1
L2/3
L5
L6
dorsal Cg1
ventral Cg2
Cingulum bundle
Induseum griseum
Medial Prefrontal Cortex (mPFC)
d
Central amygdala (CeA)
CeA
LA
CeC
CeL
CeM
EPd
BLA
Intercalated cells
Stria terminalis
e
Superior colliculus deep gray (motor)
Inferior colliculus
dPAG
lPAG
DR
vlPAG
Cuneiform nucleus
Periaqueductal gray (PAG)
C57BL/6J mice, in vivo expression verification: AAV1-hMORp1-eYFP (titer: 1.17 x 10^12 gc/mL)
f
dorsal Cg1
Medial Prefrontal Cortex (mPFC)
g
Central amygdala (CeA)
Intercalated cells
BLA
CeA
h
lPAG
Periaqueductal gray (PAG)
vlPAG
C57BL/6J mice, in vivo expression verification: AAV5-mMORp1-oScarlet (titer: 6.13 x 10^13 gc/mL)
i
Central amygdala (CeA)
BLA
CeA
j
Ventral Tegmental Area (VTA)
VTA
fr
SNr
In vivo expression validation of mMORp construct compared w/ hSyn promoter construct, C57BL/6J mice: AAV5-mMORp1-eYFP + AAV5-hSyn-mCherry (1:1)
k Central Amygdala (CeA)
BLA
hSyn-mCherry
mMORp1-eYFP
Merge
l Ventral Tegmental Area (VTA)
m Central Amygdala: hSyn+mMORp Co-labeling Quantification
Total Cell Counts
250
200
150
100
50
0
14.3%
24.1%
61.6%
37.4%
62.6%
Total=237
Total=91
mCherry (+) ONLY
eYFP (+) ONLY
mCherry/eYFP (+)
n Ventral Tegmental Area: hSyn+mMORp Co-labeling Quantification
200
150
100
50
0
22.0%
37.3%
40.7%
35.1%
64.9%
Total=354
Total=222
mCherry (+) ONLY
eYFP (+) ONLY
mCherry/eYFP (+)

Supp. Fig.2 | *mMORp1* and *hMORp1* viral constructs show robust expression in neuronal cell cultures *in vitro* and additional brain regions of interest *in vivo*, and transduce more broadly distinct populations of cells when compared to other generic promoter driven viral constructs. a-b, Transduced cultured primary cortical cell cultures from P0 rat pups with AAV1-*mMORp1*-eYFP (**a**) or AAV1-*hMORp*-eYFP (**b**). Staining for nuclei (DAPI, gray) and basal eYFP expression across cultures demonstrate the ability of both viruses to transduce neurons within culture; scale bars = 50um. **c-h,** Additional representative images from high titer/volume injections of AAV1-*mMORp1*-eYFP (**c-e**) or AAV1-*hMORp1*-eYFP (**f-h**) into putative mu opioid receptor (MOR) positive regions *in vivo* in C57BL/6J mice, including: medial prefrontal cortex (mPFC **c,f**), central amygdala (CeA, **d,g**), and ventrolateral periaqueductal gray (vlPAG, **e,h**), with basal reporter signal shown in green, and DAPI nuclei staining in blue. Restriction/overall spread of the virus within targeted regions is shown via overlaid anatomical borders on higher magnification images. L1-L6=cortical layers, Cg1= cingulate area 1, Cg2=cingulate area 2, CeC=central capsular division of the CeA, CeL=central lateral division of the CeA, CeM=central medial division of the CeA, BLA=basolateral amygdala, LA=lateral amygdala, EPd=dorsal enteropeduncular nucleus, dPAG=dorsal periaqueductal gray, lPAG=later periaqueductal gray, DR = dorsal raphe nucleus; scale bars = 500um (left), 200um (right) for **c-e**, 200um (left) and 100um (right) for **f-h**. **i-j,** Representative images of AAV1-*mMORp1*-oScalet transduced neurons in mouse CeA (**i**) and ventral tegmental area (VTA, **j**) at lower (left, scale bar = 100um) and higher (right, scale bar = 100um) magnification across both regions. SNr=substantia nigra pars reticulata, fr=fasciculus retroflexus. **k-l,** Representative images from co-injection of AAV5-*mMORp*-eYFP mixed 1μl:1μl with AAV-*hSyn*-mCherry into mouse CeA (**k**) and VTA (**l**), with expression of each fluorophore shown as a separate channel and merged on the far right. Green arrows indicate representative eYFP+ only positive cells, while red arrows indicate mCherry+ only cells, and white eYFP+/mCherry+ cells; scale bars = 100um. **m-n,** Quantification of total eYFP+, mCherry+ and eYFP+/mCherry+ cells (bar graphs), as well as average percentiles for each population of cells (middle pie graph) and eYFP+ cells overall (right pie graph) for both the CeA (n=4 ROIs from N=2 mice, **m**) and VTA (n=2, N=1, **n**).

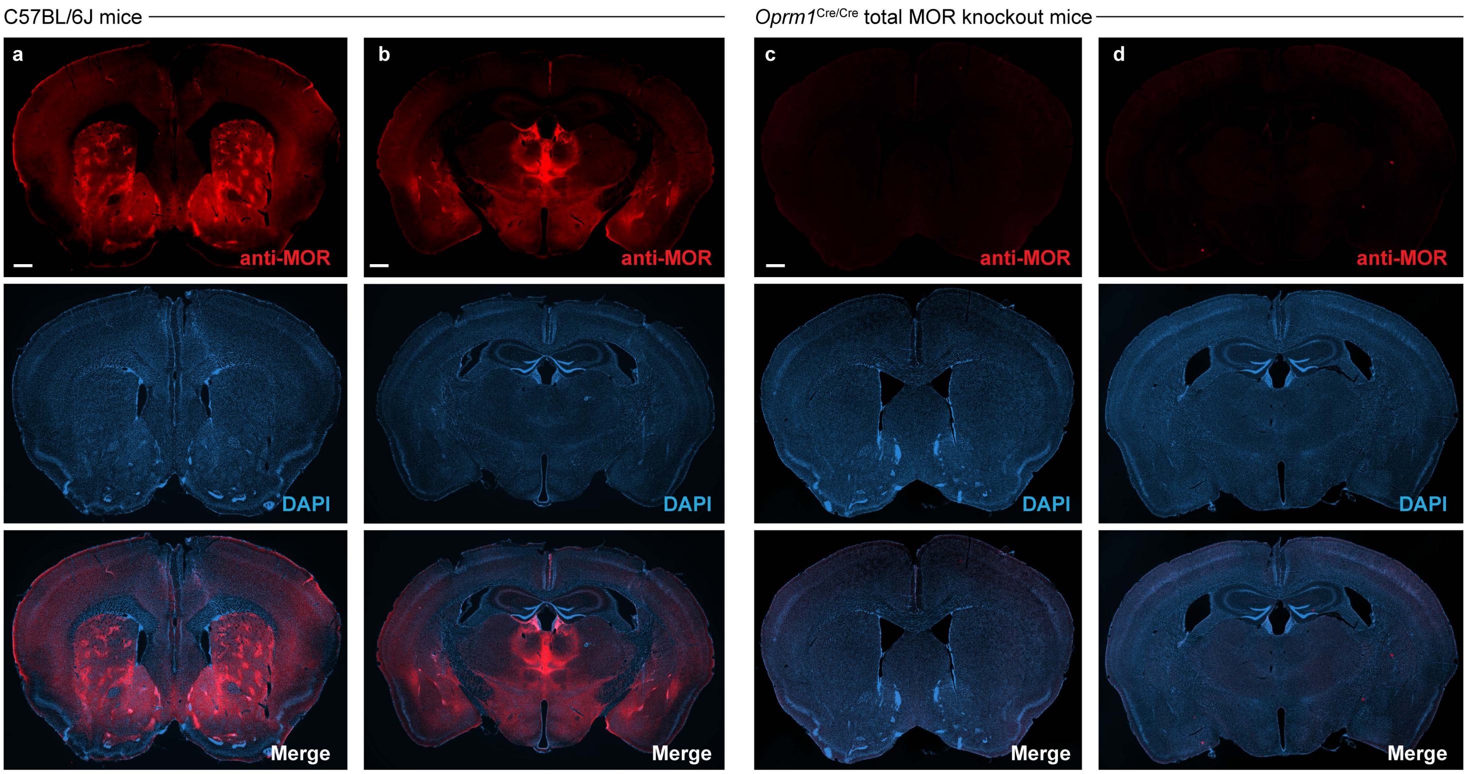

Supp. Fig. 3 | Mu opioid receptor (MOR) signal is readily detectable in MOR wildtype mice but absent in *Oprm1*$^{Cre/Cre}$ homozygous total knockouts. a-b, Representative tile scan images of tissue from C57BL/6J mice containing the dorsomedial striatum (**a**) or the medial thalamus and amygdala (**b**). Anti-MOR (Abcam, ab134054) and DAPI staining are shown as individual channel and merged to highlight the ability of this antibody to detect MOR within/around the striosomal structures in the striatum, the periventricular nucleus of the thalamus, and the central nucleus of the amygdala, regions known to harbor moderate to high levels of MOR expression. **c-d,** Similar representative images from an *Oprm1*^{Cre} animal homozygous for the Cre allele (*Oprm1*$^{Cre/Cre}$), which is known to produce a complete MOR knockout in this line. MOR detection is completely lost in the same brain regions within this mouse when using the same antibody stain. Scale bars = 500um.

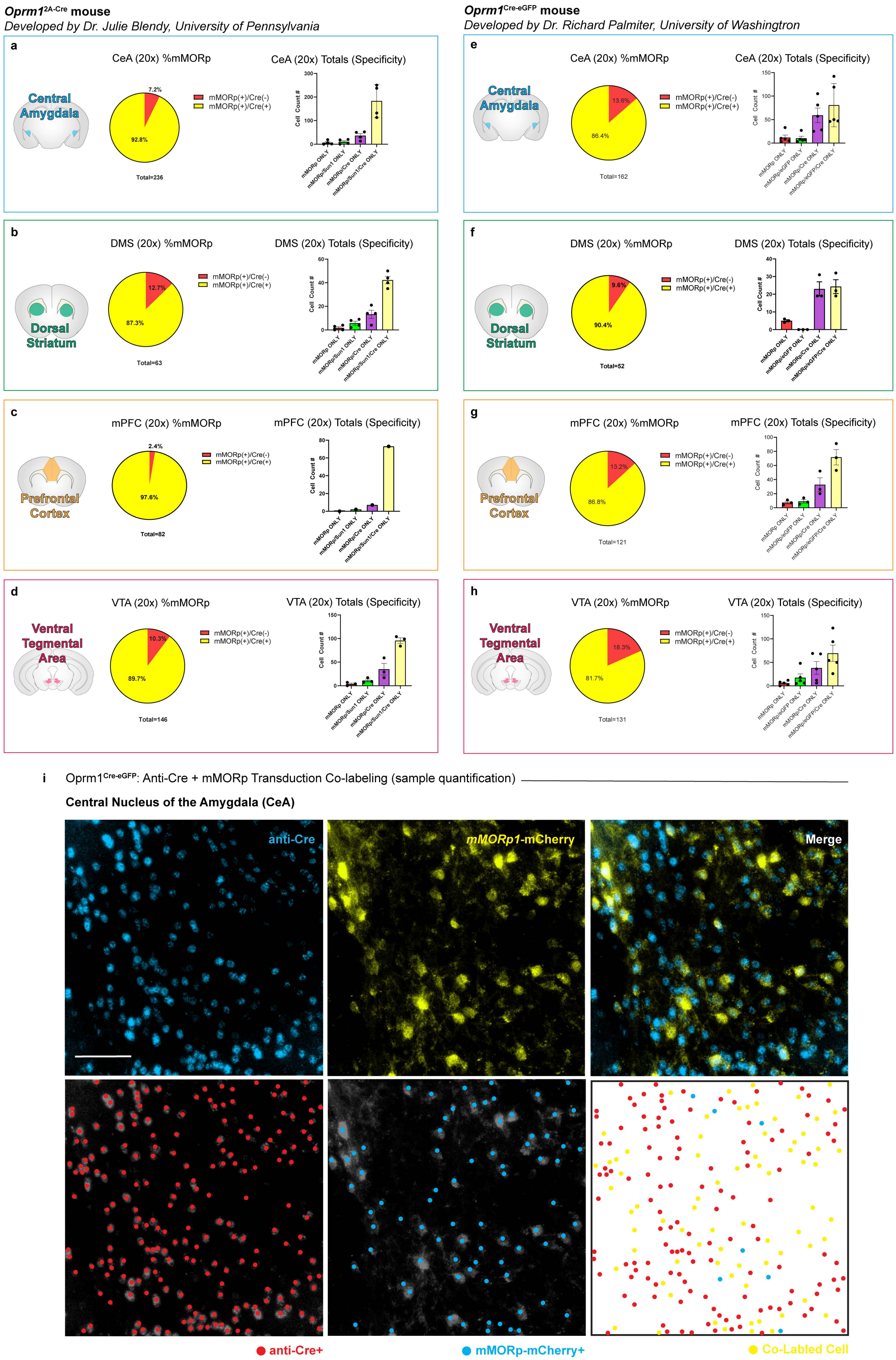
Oprm1 2A-Cre mouse
Developed by Dr. Julie Blendy, University of Pennsylvania
Oprm1 Cre-eGFP mouse
Developed by Dr. Richard Palmiter, University of Washingtron
a
CeA (20x) %mMORp
CeA (20x) Totals (Specificity)
Central Amygdala
7.2%
92.8%
Total=236
mMORp(+)/Cre(-)
mMORp(+)/Cre(+)
Cell Count #
mMORp ONLY
mMORp/Sun1 ONLY
mMORp/Cre ONLY
mMORp/Sun1/Cre ONLY
b
DMS (20x) %mMORp
DMS (20x) Totals (Specificity)
Dorsal Striatum
12.7%
87.3%
Total=63
c
mPFC (20x) %mMORp
mPFC (20x) Totals (Specificity)
Prefrontal Cortex
2.4%
97.6%
Total=82
d
VTA (20x) %mMORp
VTA (20x) Totals (Specificity)
Ventral Tegmental Area
10.3%
89.7%
Total=146
e
CeA (20x) %mMORp
CeA (20x) Totals (Specificity)
Central Amygdala
13.6%
86.4%
Total=162
mMORp/eGFP ONLY
mMORp/eGFP/Cre ONLY
f
DMS (20x) %mMORp
DMS (20x) Totals (Specificity)
Dorsal Striatum
9.6%
90.4%
Total=52
g
mPFC (20x) %mMORp
mPFC (20x) Totals (Specificity)
Prefrontal Cortex
13.2%
86.8%
Total=121
h
VTA (20x) %mMORp
VTA (20x) Totals (Specificity)
Ventral Tegmental Area
18.3%
81.7%
Total=131
i
Oprm1 Cre-eGFP: Anti-Cre + mMORp Transduction Co-labeling (sample quantification)
Central Nucleus of the Amygdala (CeA)
anti-Cre
mMORp1-mCherry
Merge
anti-Cre+
mMORp-mCherry+
Co-Labled Cell

Supp. Fig. 4 | Quantification of *mMORp*-mCherry+ cell populations observed in the CeA, DMS, mPFC and VTA of *Oprm1*$^{2A\text{-}Cre}$ and *Oprm1*^{Cre} mice. a-d, Average counts of total *mMORp*-mCherry positive cell populations quantified across all four regions of interest from viral specificity validation studies performed in *Oprm1*$^{2A\text{-}Cre}$:Sun1 reporter mice injected with AAV1-*mMORp*-hM4Di-mCherry, with percentile averages shown in parts of a whole charts on the left, and averages for individual cell population counts tabulated in bar graphs on the right. Population counts were performed by counting all cells positive in designated ROIs for *mMORp*-mCherry expression, as well as all those positive for mCherry and anti-Cre staining or Sun1-GFP reporter alone or together, with results for the CeA (~92.8%, N=2 mice, n=4 ROIs, **a**), DMS (~87.3%, N=2, n=4, **b**), mPFC (~97.6%, N=1, n=1, **c**) and VTA (~89.7%, N=2, n=3, **d**) displayed. **e-h,** Similar graphs and charts of averaged population counts and percentiles for *mMORp*-mCherry+ cells in the CeA (~86.4%, N=3 , n=5, **e**), DMS (~90.4%, N=3 ,n=3, **f**), mPFC (~86.8%, N=2 , n=3, **g**) and VTA (~81.7%, N=3, n=5, **h**) of *Oprm1*^{Cre} mice co-injected with a mix of AAV1-*mMORp*-hM4Di-mCherry and AAV5-*hSyn*-DIO-eGFP viruses in each region of interest. Population counts were performed by counting all cells positive in designated ROIs for *mMORp*-mCherry and eGFP expression, as well as all those positive for mCherry or eGFP and anti-Cre staining or Sun1-eGFP reporter alone or together. *mMORp*-mCherry+/anti-Cre+ cell populations from both lines were combined to perform the analyses discussed and presented in **Fig. 1n**. **i,** Example of an ROI from the CeA used for quantifying anti-Cre+ and *mMORp*-mCherry+ cells. Upper images show signal for both stain as separate channels and merged to overlay one another, while lower images show the same stains in greyscale overlaid with markers used to designate either anti-Cre+ (red) or *mMORp*-eYFP+ (blue) cells. Marker maps are shown merged on the bottom right, with Cre+/eYFP+ cells (yellow) displayed against Cre+/eYFP- and Cre-/eYFP+ cells within the given ROI. Scale bar=200um.

Glial Marker Analysis Panel: C57BL/6J mice: AAV1-*mMORp1*-eYFP (titer: 6.90 x 10^{12} - 6.90 x 10^{11} gc/mL)

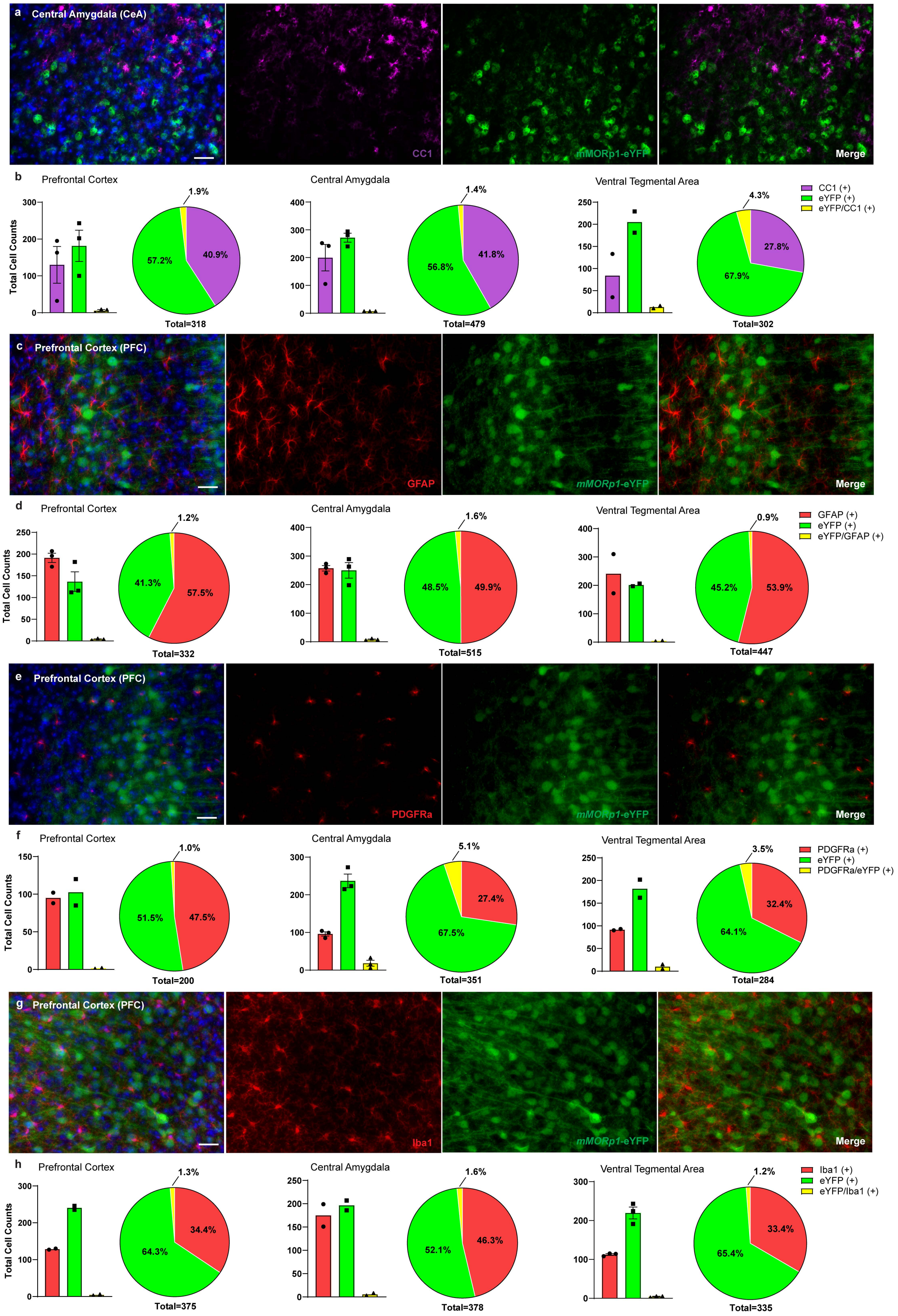

Supp. Fig. 5 | *mMORp*-eYFP signal within the mPFC, CeA and VTA of mice shows little to no overlap on cells labeled for astrocytic, oligodendritic or microglial markers. a-b, Results of staining and quantification for the overlap of the oligodendrocyte glial cell marker, CC1, observed on DAPI delineated cells positively transduced with *mMORp*-eYFP within the mouse mPFC, CeA and VTA. High magnification representative images from the CeA (**a**) show antibody amplified signal for both marker and fluorophore separately and overlaid. Quantification of total CC1+, eYFP+ and CC1+/eYFP+ cells across all three brain regions are shown as bar graphs on the right, while total percentiles for each population are presented in pie graphs on the left, with the percent of CC1+/eYFP+ cells shown (mPFC: 1.9%, n=3 ROIs from N=2 mice; CeA: 1.4%, n=3, N=2; VTA: 4.3%, n=2, N=1; **b**). **c-d,** Similar images and quantification results are shown for staining performed with antibodies for the astrocytic marker GFAP, showing representative images from the mPFC (**c**) and percentiles for GFAP+/eYFP+ cell counts across regions (mPFC:1.2%, n=3, N=2; CeA: 1.6%, n=3, N=2; VTA: 0.9%, n=2, N=1; **d**). **e-f,** Representative images from the mPFC for stained for the oligodendrocyte precursor marker, PDGFRα (**e**) are shown above total cell counts and PDGFRα+/eYFP+ percentile quantification results (mPFC: 1.0%, n=2, N=1; CeA: 5.1%, n=3, N=2; VTA: 3.5%, n=2, N=1; **f**). **g-h,** Similar representative images (from the mPFC as well, **g**) and quantification of the total percentage of cells positive for the microglial marker Iba1 and eYFP are shown in the lowest panel (mPFC: 1.3%, n=2, N=1; CeA: 1.6%, n=2, N=1; VTA: 1.2%, n=3, N=2; **h**). Scale bars across all images=100um.

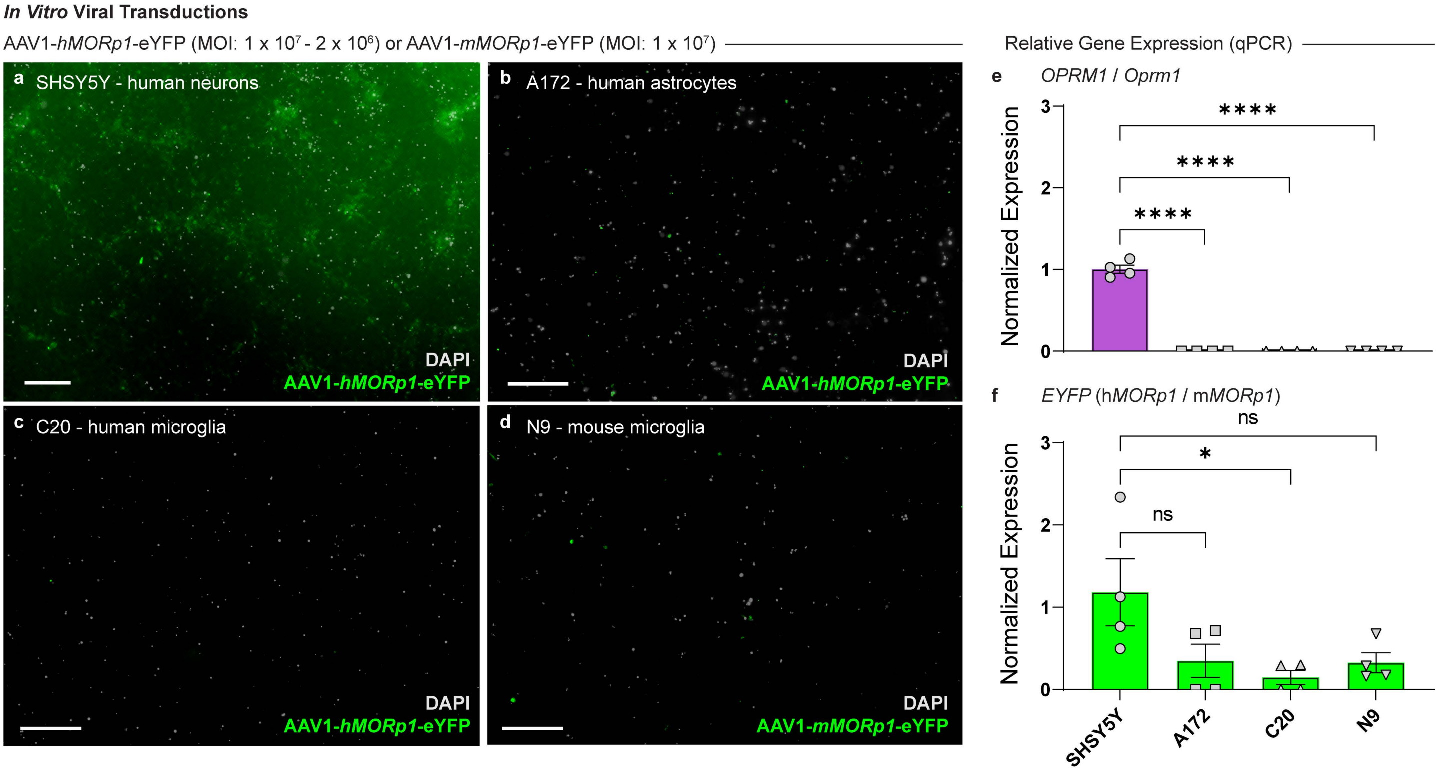

Supp. Fig. 6 | Human and murine cultured microglia and astrocyte cell lines show little to no gene expression for *OPRM1* or *Oprm1* in comparison to cultured neuronal cells, and reduced *EYFP* expression following transduction with *hMORp*-eYFP or *mMORp*-eYFP. a-d, Representative, high magnification images of individual wells from plates of cultured human SHSY5Y neurons (**a**), human A172 astrocytes (**b**) and human C20 microglia (**c**) transduced with the *hMORp*-eYFP viral construct (1×10^{11} gc/mL viral genomic content per well, MOI: 2×10^6 [SHSY5Y] & 1×10^7 [A172 & C20]), as well as mouse N9 microglia (**d**) transduced with the *mMORp*-eYFP viral construct (1×10^{11} gc/mL, MOI: 1×10^7) are shown on the left. Scale bars for all images=100um. **e-f,** Summary data of the relative gene expression for both *OPRM1* (in human lines) or *Oprm1* (in murine line) and *EYFP* are shown on the right. Expression for genes of interest across lines is normalized to the house keeping gene GAPDH, and all normalized expression values are displayed as relative to the expression of either *OPRM1*/*Oprm1* or *EYFP* in SHSY5Y cells. Normalized expression of *OPRM1* and *Oprm1* was found to be low in A172 human astrocytes (0.00051, n=4), C20 human microglia (0.00039, n=4) and N9 mouse microglia, respectively (0.000076, n=4), while human neuronal SHSY5Y cultures showed relatively higher OPRM1 expression overall (1.004, n=4), with one way ANOVA analyses and Dunnett's post hoc tests revealing SHSY5Y expression to be significantly great than that of all other cells lines tested (main effects: P<0.0001 [cell line x gene expression]; multiple comparisons: SHSY5Y v. A172, P<0.0001, SHSY5Y v. C20, P<0.00001, SHSY5Y v. N9, P<0.0001). Similarly, normalized *EYFP* expression was found to be highest in the SH-SY5Y cells (1.182, n=4) and lower by comparison in human A172 astrocytes (0.350, n=4), human C20 microglia (0.147, n=4) and mouse N9 microglia (0.325, n=4), with one way ANOVA analyses and Dunnett's post hoc tests showing *EYFP* to be significantly different across cell lines in general, and lower in the C20 line overall (main effects: P=0.0409 [cell line x gene expression]; multiple comparisons: SHSY5Y v. A172, P=0.0730, SHSY5Y v. C20, P=0.0251, SHSY5Y v. N9, P=0.0645). All data in graphs are presented as averages ± the SEM. MOI = multiplicities of infections.

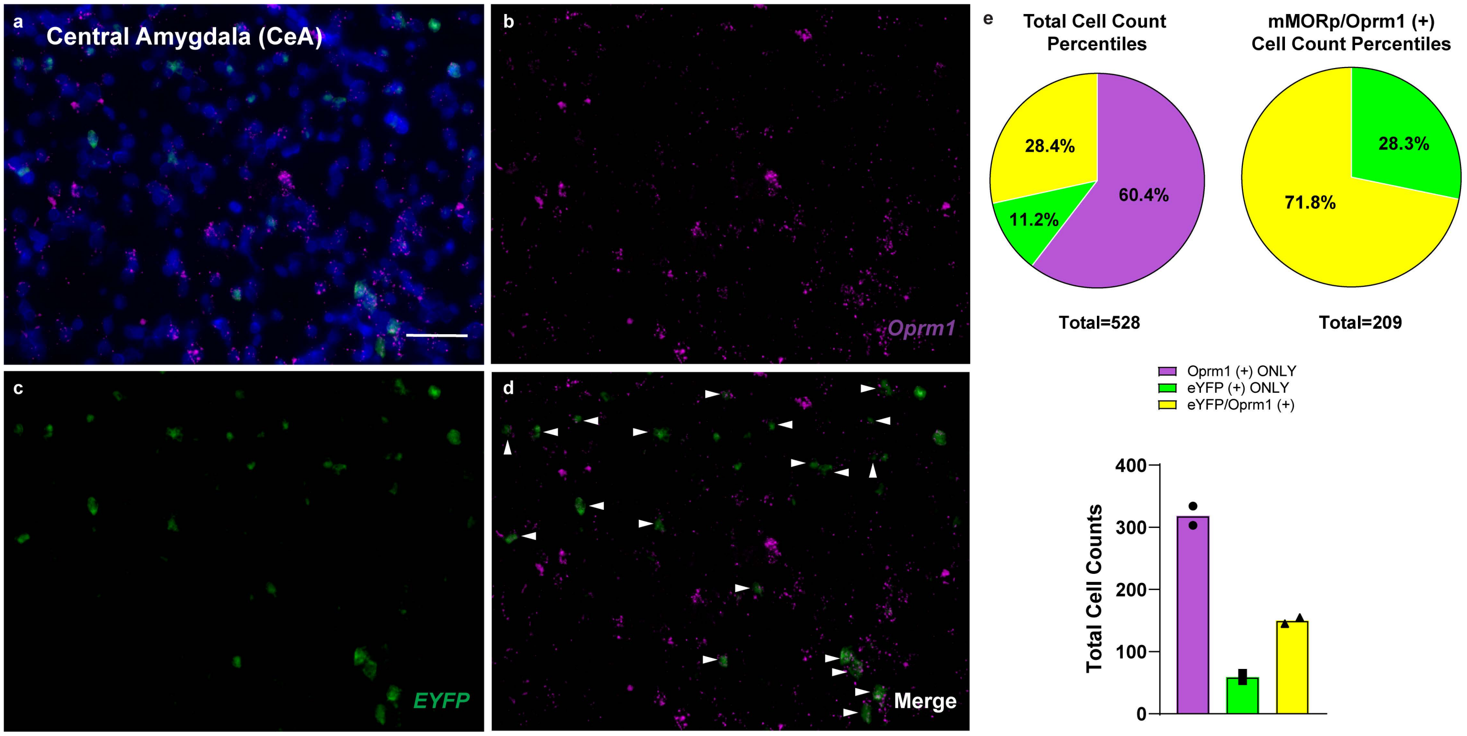

Supp. Fig. 7 | *mMORp*-eYFP transduced neurons in rat tissue show robust co-labeling for *Oprm1* and *EYFP* mRNA transcripts. a-d, Representative, high magnification images of rat CeA tissue prepared for FISH following intracranial injection of the *mMORp*-eYFP construct. Signal for mRNA transcript probes targeted to endogenous rat *Oprm1* and virally transduced *EYFP* are shown overlaying DAPI stained cellular nuclei (**a**), individual as separate channels for both *Oprm1* (**b**) and *EYFP* (**c**) and overlaid (**d**) to demonstrate the degree of co-localization observed for both transcript markers on individual neurons. White arrows indicate representative *EYFP+*/*Oprm1+* cells. Scale bars for all images=100um. **e,** Average percentiles and quantification for the total number of *Oprm1*+, *EYFP*+ and *EYFP+*/*Oprm1*+ counted across selected ROIs of high magnification images of the rat CeA are presented on the right as pie graphs (upper) and bar graphs (lower), respectively. *EYFP+*/*Oprm1*+ neurons were found to comprise the majority of all *EYFP*+ cells counted across CeA ROIs (71.8%, n=2 ROIs from N=1 rat). Data in bar graphs are presented as averages ± SEM.

Glial Marker Panel Analysis: Rat & Shrew

Rat — Sprague-Dawley — AAV1-*mMORp1*-eYFP (titer: 6.9 x 10^{11} gc/mL)

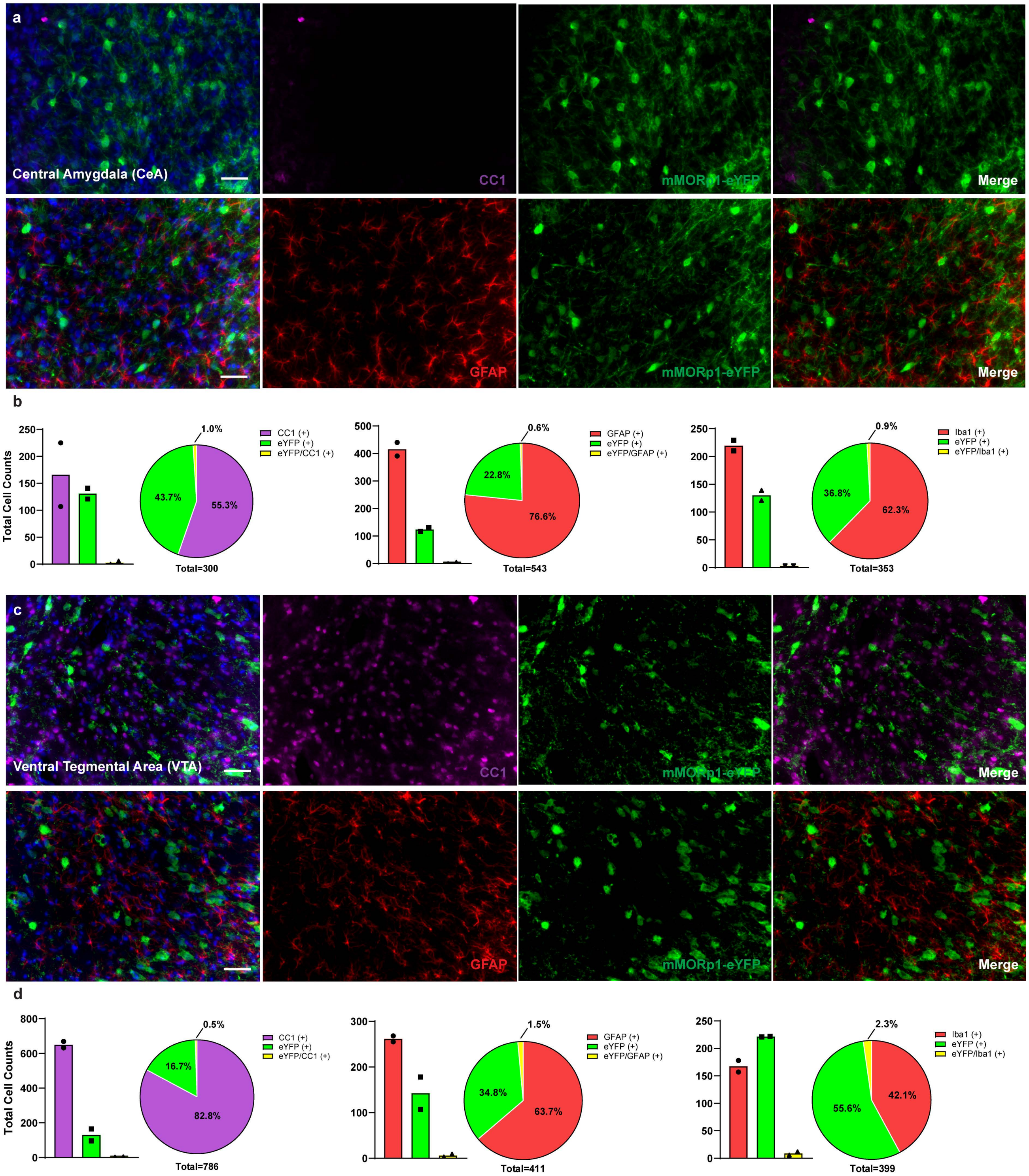

Asian House Shrew (*Suncus Murinus*) — AAV1-*mMORp1*-eYFP (titer: 6.9 x 10^{11} gc/mL)

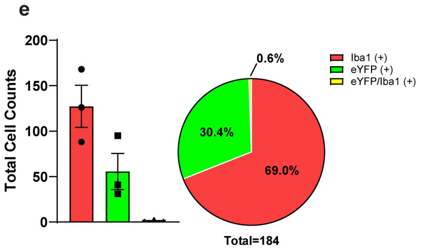

Supp. Fig. 8 | *mMORp*-eYFP signal within the CeA and VTA of rats, and the AP/NTS of shrews, shows little to no overlap on cells labeled for astrocytic, oligodendritic or microglial markers. a-b, Results of staining and quantification examining the overlap of cells positively stained for CC1 (oligodendrocytes), GFAP (astrocytes) and Iba1 (microglia) with virally transduced eYFP within the rat CeA. High magnification representative images (**a**) show antibody amplified signal for CC1 (upper) and GFAP (lower) markers, as well as *mMORp*-eYFP, individually and overlaid, complementing representative images for Iba1 staining shown in this region in **Fig. 2.** Quantification of CC1, GFAP and Iba1 cell populations are shown below as bar graphs on the right, while total percentiles for each population are presented in pie graphs on the left (CC1: 1.0% CC1+/eYFP+, n=2 ROIs from N=1 rat; GFAP: 0.6% GFAP+/eYFP+, n=2, N=1; Iba1: 0.9% Iba1+/eYFP+, n=2, N=1; **b**). **c-d,** Similar images and quantification results are shown for staining performed within rat VTA, with representative images (**c**) shown for the successfully staining of CC1 (upper) and GFAP (lower) in rat tissue, complementing previously shown Iba1 staining results (**Fig. 2**). Quantification of CC1, GFAP and Iba1 cell populations are shown below as bar graphs on the right, while total percentiles for each population are presented in pie graphs on the left (CC1: 0.5% CC1+/eYFP+, n=2, N=1; GFAP: 1.5% GFAP+/eYFP+, n=2, N=1; Iba1: 2.3% Iba1+/eYFP+, n=2, N=1; **d**). **e,** Quantification of Iba1+/eYFP+ cell counts in the shrew AP/NTS shown as bar graphs on the right, and as total percentiles for each population in pie graphs on the left, with representative images of successful Iba1 staining in this structure shown previously in **Fig. 2** (Iba1: 0.6% Iba1+/eYFP+, n=3, N=2). As staining for PDGFRα in rat tissue, and CC1, GFAP and PDGFRα in shrew tissue provided unsuccessful, these data were not shown or quantified. Scale bars across all images=100um.

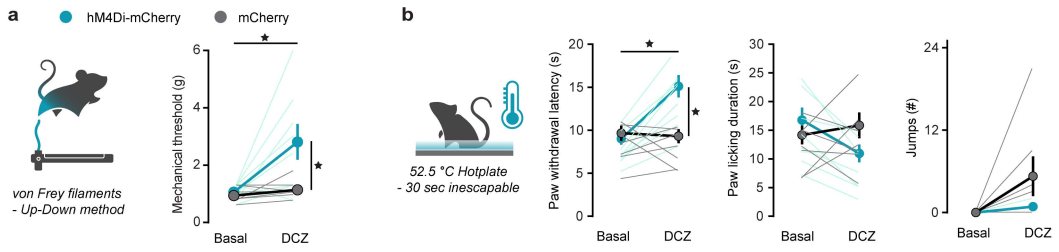

Supp. Fig. 9 | Activation of *mMORp*-hM4Di in spinal cord neurons via intrathecal deschloroclozapine (DCZ) produces analgesic effects similar to CNO treatment. a, Mechanical sensory thresholds (von Frey Up-Down testing) in *mMORp*-hM4Di-mCherry injected mice (N=9 mice) compared with *hSyn*-mCherry injected controls (N=7 mice) at baseline and 30 minutes following intrathecal DCZ administration (10 pg; Two-way ANOVA + Bonferroni: main effect: P=0.033 [viral treatment x DCZ treatment], P=0.011 [DCZ treatment], P=0.030 [viral treatment]; multiple comparisons: basal v. DCZ, P>0.999 [basal], P=0.004 [DCZ]). Average response changes per group shown as thick gray (mCherry) or blue (hM4Di) lines. Individual mice are shown as thin gray and blue lines. **b,** Nocifensive behaviors on inescapable 52.5°C hot plate over a 30-sec trial: latency (sec) to hind paw withdrawal (two-way ANOVA + Bonferroni: main effect: P=0.0003 [viral treatment x DCZ treatment], P=0.0007 [DCZ treatment]; multiple comparisons: basal v. DCZ, P>0.999 [basal], P=0.0005 [DCZ]), hind paw licking duration (two way ANOVA + Bonferroni; main effects: P=0.022 [viral treatment x DCZ treatment]; multiple comparisons: basal v. DCZ, P=0.693 [basal], P=0.170 [DCZ]), and total jumping bouts (two way ANOVA + Bonferroni; main effects: P=0.045 [DCZ treatment]; multiple comparisons: basal v. DCZ, P>0.999 [basal], P=0.067 [DCZ]). All data are presented as means ± SEM, *P<0.05.

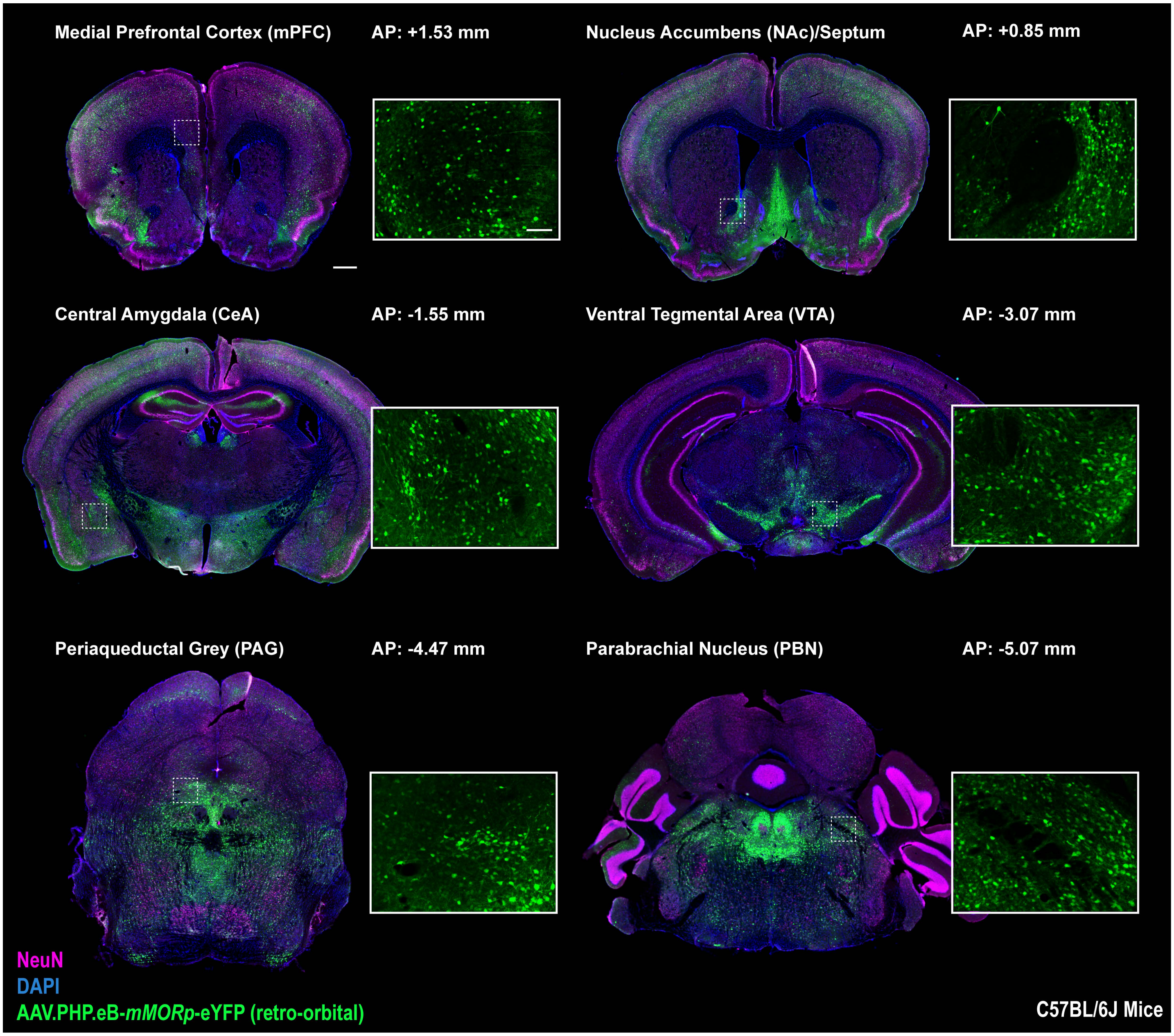

Supp. Fig. 10 | PHP.eB-*mMORp*-eYFP transduction is visible in putative MOR+ structures throughout the brain. CNS expression in representative coronal sections from the brains of C57BL/6J mice injected retro-orbitally with the AAV.PHP.eB-*mMORp*-eYFP virus, with representative MOR+ structures containing within each section indicated along the anterior-posterior axis (relative to Bregma) and shown at higher magnification in insets presented next to each low magnification image of an individual section. Scale bars for whole tissue sections=1000um and for insets=200μm.

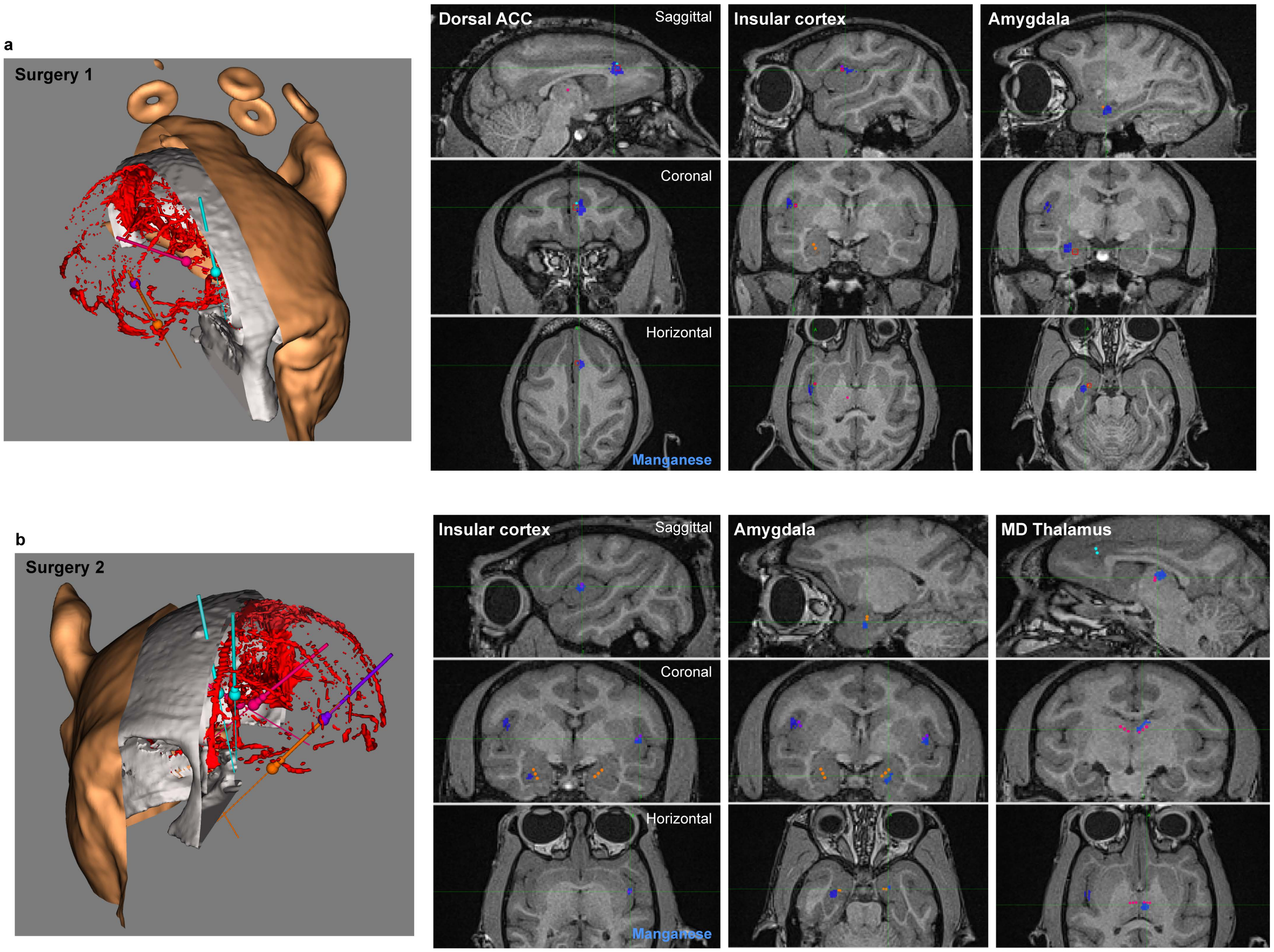

Supp. Fig. 11 | Macaque intracranial injection targeting vectors and post-operative imaging. a-b, 3D reconstructions of the macaque skin (tan), skull (white) and vasculature (red) from T1 MRI along injection trajectories calculated to deliver both *mMORp* and *hMORp*-eYFP viruses to the anterior cingulate cortex (blue), insular cortex (purple), mediodorsal thalamus (pink) and amygdala (orange) across the first (top, **a**) and second (bottom, **b**) surgical procedures performed on the subject (left). Images from post-operative manganese-enhanced MRI scans performed to validate injection accuracy are shown on the right for each targeted region across three planes of view. Manganese contrast signal (shown in light blue, 100mM solution mixed 1:100 with each virus) serves as an *in vivo* marker of where each virus was delivered relative to the planned injection sites (colored dots).

Anterior Cingulate Cortex — AAV1-*hMORp1*-eYFP (titer: 1.17 x 10^{12} gc/mL)

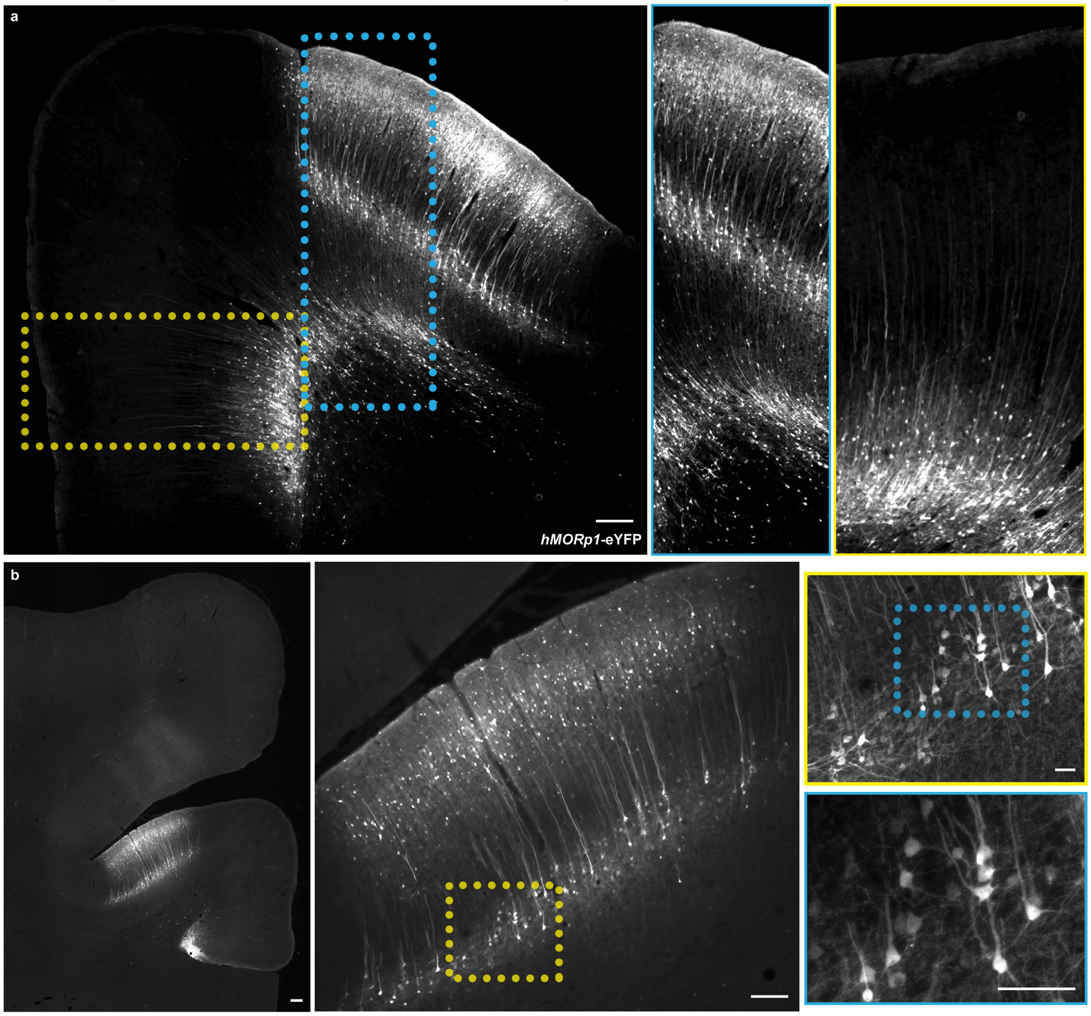

Anterior Insular Cortex — AAV1-*hMORp1*-eYFP (titer: 1.17 x 10^{12} gc/mL)

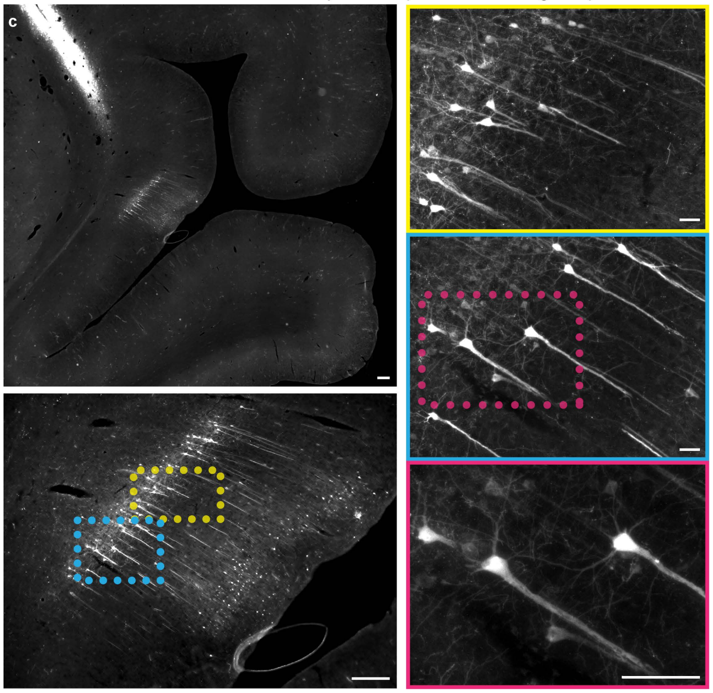

Amygdala — AAV1-*mMORp1*-eYFP (titer: 1.40 x 10^{13} gc/mL)

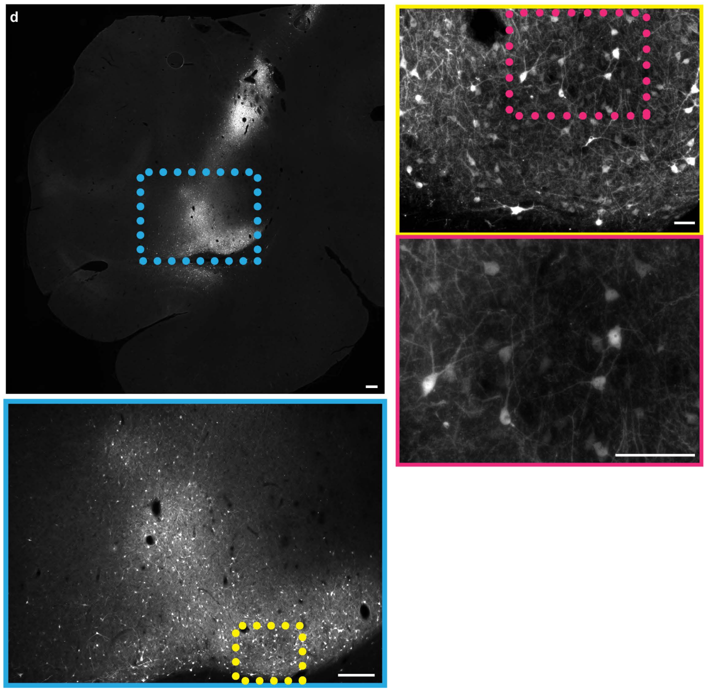

Supp. Fig. 12 | Visualization of *hMORp*-eYFP viral expression within the macaque anterior cingulate cortex and insular cortex, and *mMORp*-eYFP within the amygdala. a, Additional low (left) and high (right) magnification images of macaque dorsal anterior cingulate cortex (dACC) tissue following injection of the *hMORp*-eYFP virus. Expression of eYFP reporter tagged cell bodies (antibody staining enhanced, white) can be noted across cortical layers (blue boxed region), and processes extending up from cortical layer VI neurons into the upper cortical layers can also be observed (yellow boxed regions). Scale bar = 200um (left), right = digital zoom on boxed regions. **b,** Low and high magnification imaging of transduced cell bodies within layer V of the dACC. Scale bars = 1000um (left), 200um (middle), 100um (yellow box), 100um (blue box). **c,** Additional low and high magnification images of macaque insular cortex tissue following injection of the *hMORp*-eYFP virus. Processes and cell bodies of eYFP transduced neurons (antibody staining enhanced, white) within layer Vb are shown at both lower magnification (left, scale bars = 1000um [upper], 200um [lower]) and higher magnification (right, scale bars = 100um [yellow and blue boxes], 100um [pink box]). **d,** Additional images of macaque amygdala following *mMORp*-eYFP transduction. Visualization of transduced cell bodies and processes (white) can be noted at lower magnificaiton (left, scale bars = 1000um [upper], 200um [lower]), and at higher magnification (right), including detailed imaging of these individual bodies and processes (scale bars = 100um [yellow box], 100um [pink box]).

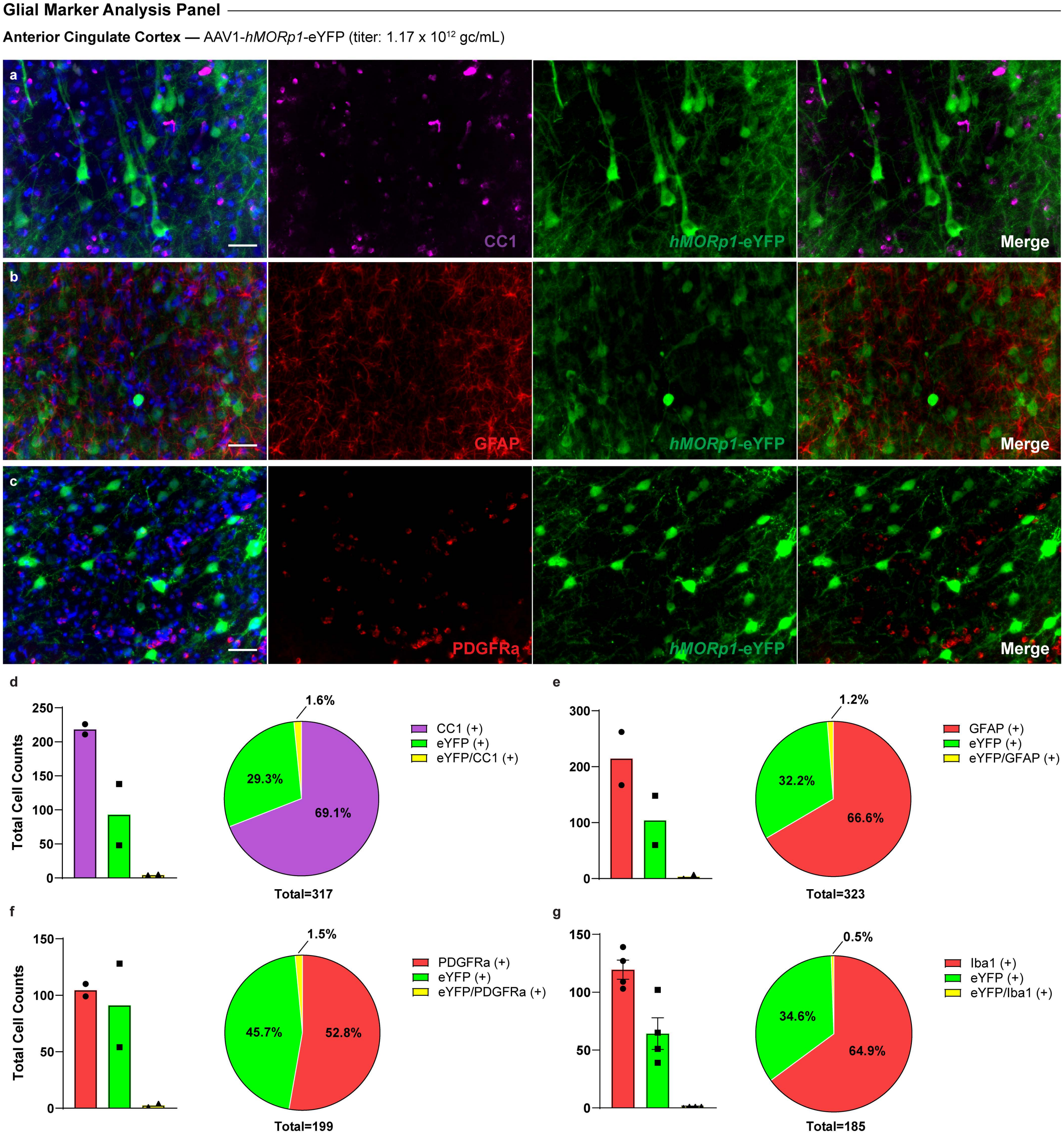

Supp. Fig. 13 | *hMORp*-eYFP signal within the dACC of rhesus macaque shows little to no overlap on cells labeled for astrocytic, oligodendritic or microglial markers. a-b, Results of staining and quantification examining the overlap of cells positively stained for CC1 (oligodendrocytes), GFAP (astrocytes), PDGFRα (oligodendrocyte precursors), or Iba1 (microglia) with virally transduced eYFP within the macaque dACC. High magnification representative images show antibody amplified signal for CC1 (**a**), GFAP (**b**) and PDGFRα (**c**) markers, as well as *hMORp*-eYFP signal, separately and overlaid, complementing representative images for Iba1 staining shown for this region in **Fig. 6. d-g,** Quantification of CC1+/eYFP+ (**d**), GFAP+/eYFP+ (**e**), PDGRFα+/eYFP+ (**f**) and Iba1+/eYFP+ (**g**) cells are shown below as bar graphs on the right, while total percentiles for each population are presented in pie graphs on the left (CC1: 1.6%, n=2 ROIs from N=1 macaque; GFAP: 1.2%, n=2, N=1; PDGFRα: 1.5%, n=2, N=1; Iba1: 0.5%, n=2, N=1). Scale bars across all images=100um.

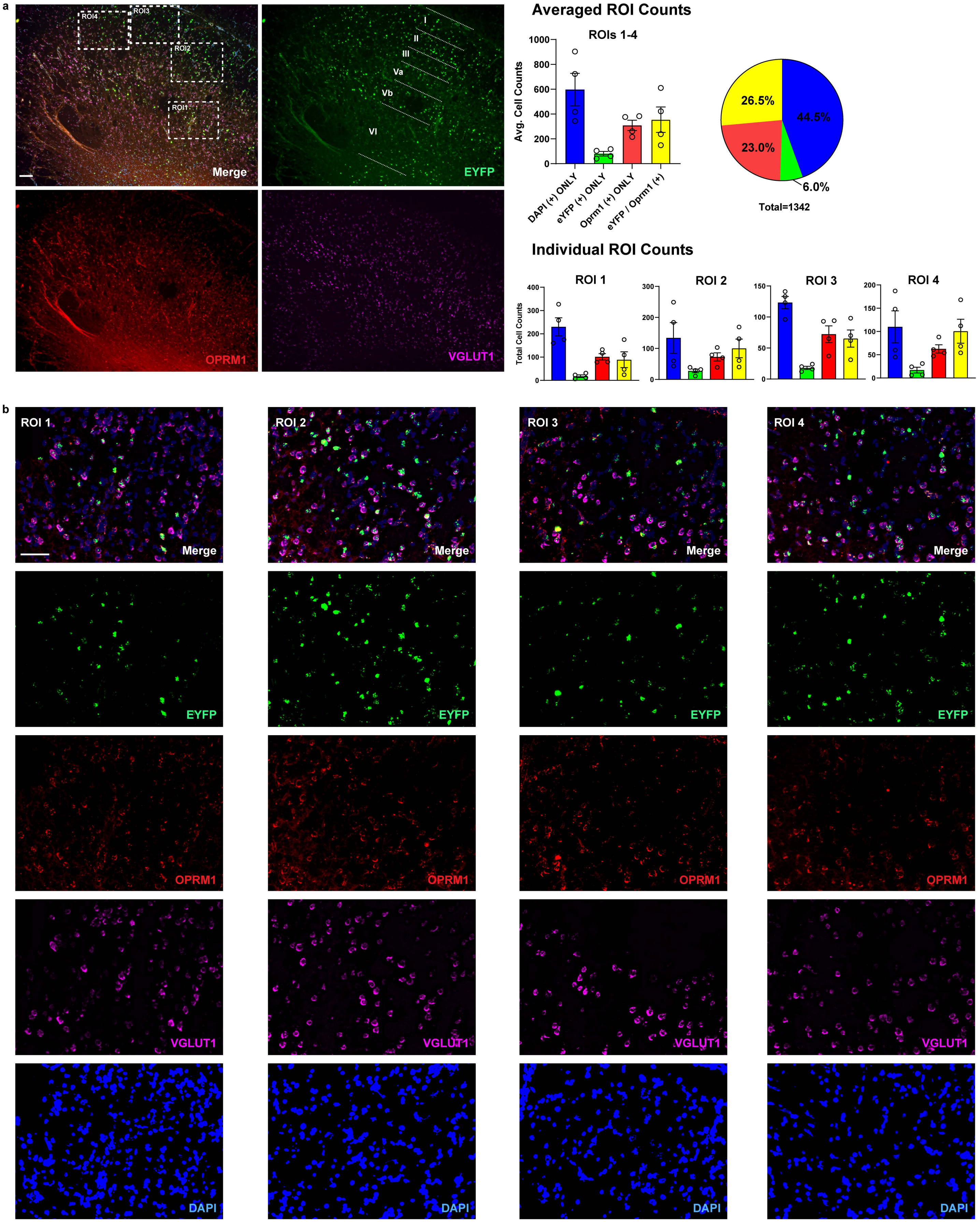
a
ROI4
ROI3
ROI2
ROI1
Merge
I
II
III
Va
Vb
VI
EYFP
OPRM1
VGLUT1
Averaged ROI Counts
ROIs 1-4
Avg. Cell Counts
DAPI (+) ONLY
eYFP (+) ONLY
Oprm1 (+) ONLY
eYFP / Oprm1 (+)
26.5%
44.5%
23.0%
6.0%
Total=1342
Individual ROI Counts
ROI 1
ROI 2
ROI 3
ROI 4
Total Cell Counts
b
ROI 1
ROI 2
ROI 3
ROI 4
Merge
EYFP
OPRM1
VGLUT1
DAPI

Supp. Fig. 14 | Quantification of *hMORp*-eYFP transduced *OPRM1*+ cells within macaque anterior cingulate cortex. a, Lower magnification images (upper left) of macaque tissue containing the dorsal anterior cingulate cortex (dACC) processed for RNAscope FISH in order to visualize cells positive for *OPRM1, EFYP* or *VGLUT1* mRNA transcript expression. Cortical layer borders are shown to demonstrate the spread of positively transduced neurons within the macaque dACC following a single injection of the *hMORp*-eYFP virus (upper right). Graphs tabulating the average and individual total cells labeled for *OPRM1* and *EYFP* transcript counted within four representative regions of interest (ROI1-4) are shown on the right. Averaged ROI counts represent the combined scoring from four independent experimenters analyzing each ROI for the single or co-labeled groups of interest designated in the bar graph. Individual ROI counts show the total counts for each of these groups made by the experimenters across each ROI separately. Scale bar = 100um. **b,** Merge and single channel higher magnification images from ROIs 1-4 used to produce the counts tabulated above. All cell bodies denoted by DAPI staining were counted and assessed for both labeling and co-labeling for all transcripts of interest within the boundaries of each 20x ROI image. Scale bar = 100um. All data in bar graphs are presented as means ± SEM.

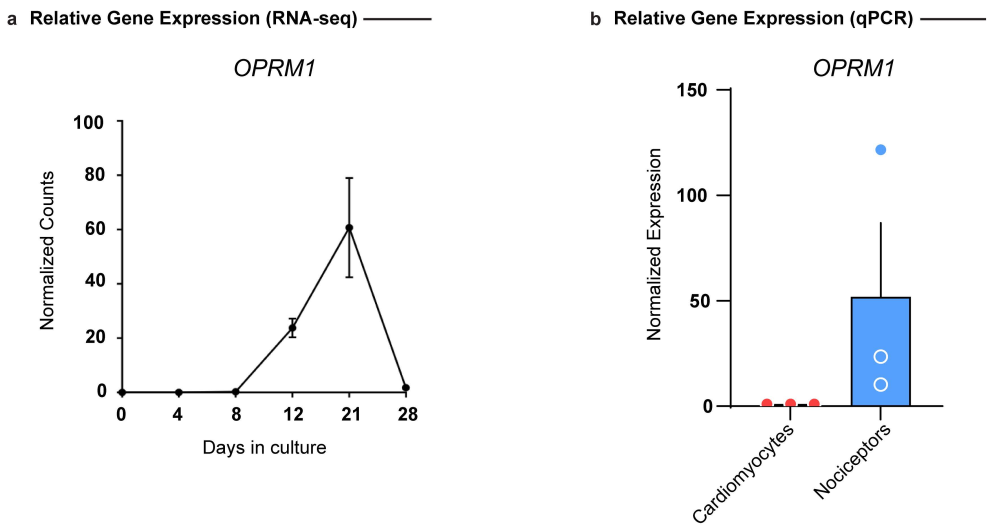

Supp. Fig. 15 | Human iPSC-derived nociceptors show evidence of gene expression for *OPRM1* in comparison to cultured human cardiomyocytes. Summary data of the relative gene expression for *OPRM1* in nociceptor and cardiomyocyte cells cultured and differentiated from human iPSCs (LiPSC-GR1.1 line). a, Transcriptomic data demonstrating the relative expression level of *OPRM1* observed in iPSC-derived nociceptor-like cells. Expression levels (displayed as normalized transcript counts) for *OPRM1* are shown over the course of 28 days during cell differentiation, with cells collected and analyzed at different time points. b, *OPRM1* relative expression from qPCR studies conducted with cultured cardiomyocyte and nociceptor cell types differentiated from the LiPSC-GR1.1 line. Expression is normalized to the house keeping gene GAPDH, and all normalized values are displayed as relative to the expression of *OPRM1* in the cardiomyocytes. Averaged, normalized expression of *OPRM1* in cultured nociceptors was found to trend higher than that of the cardiomyocytes (51.83, n=3 [nociceptors]; 1, n=3 [cardiomyocytes]; two-tailed, unpaired student's t-test: P=0.2215), indicating a basal level of expression of *OPRM1* in these cell types around the 21 day time point at which they were harvested. All data in graphs are presented as averages ± the SEM.

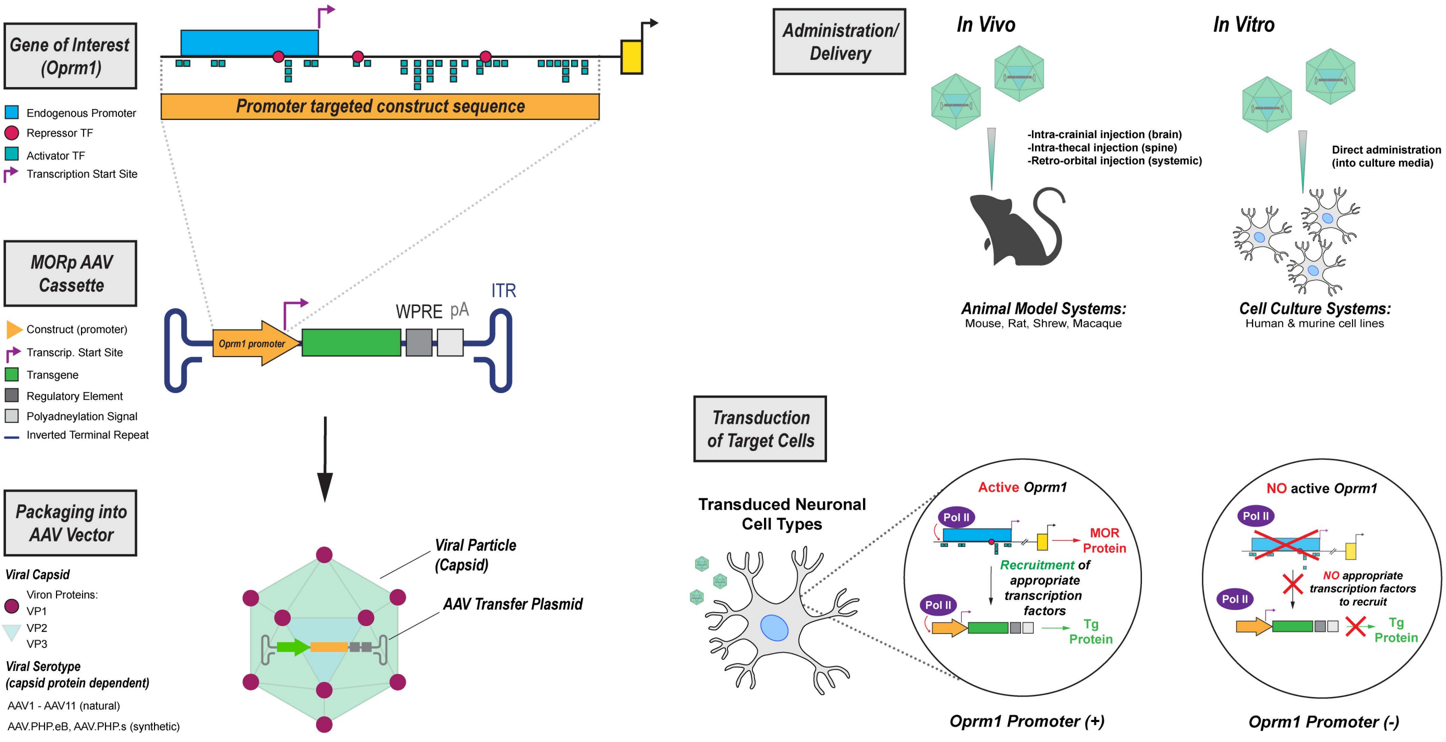

Supp. Fig. 16 | Design, packaging, routes of administration and expression mechanisms of the *MORp* constructs. *Upper left:* Coding sequence for the *Oprm1* gene & complementary sequence designed for the *MORp* constructs matching this region, with icons indicating the endogenous *Oprm1* promoter region, binding areas for both repressor and activator transcription factors, and putative transcription start sites. ***Middle left:*** AAV cassette design for the *MORp* constructs, which is composed of: custom sequence designed to be complementary to the *Oprm1* promoter, TSS sequence sequence, transgene element driven by the promoter, and regulatory elements. ***Lower left:*** The cassette, contained within an AAV transfer plasmid, can be packaged into different variants of AAV depending on the specific serotype desired. AAV serotype is determined by the unique antigenicity and surface structure of the viral proteins (VP1-3) that comprise the viral particle's outer layer (capsid). Different conformations of the capsid, as well as capsid protein interactions, define each serotype, and impart on them different levels of selectivity for the tissue and cell types they can most successfully transduce. AAV serotypes 1-11 are naturally occurring, while synthetic variants such as AAV.PHP.eB or AAV.PHP.s have been produced using AAV directed evolution platforms. ***Upper right:*** Packed AAVs can be introduced into tissue or cell types of interest via multiple routes of administration. For *in vivo* applications, AAVs can be administered directly via injection using a syringe to deliver virus ei-ther focally within a single tissue type/region of interest or systemically throughout the entire body. For *in vitro* applications, admin-istration of virus directly onto cells or tissue within culture is usually sufficient. Viral particles will then be able to bind to cells by tar-geting surface receptors along their membranes, enter said cells, and release their genomic material. ***Lower right:*** as AAV are usually not integrating, exogenous viral DNA will instead co-opt the necessary transcriptional machinery present within the cells. For the *MORp* constructs, transcription will only occur in cells in which a unique combination of transcription factors are able to bind to the native *Oprm1* promoter and recruit additional transcriptional machinery (i.e. polymerases like poll II) necessary to transcribe the *Oprm1* gene to produce it's cognate mRNA transcripts. Cells transduced by AAV-*MORp* that contain active endogenous *Oprm1* pro-moter will also transcribe the virally introduced *MORp* construct. By contrast, cells that do not possess an active *Oprm1* promoter, even if they are transduced by the virus, will not be able to recruit the necessary transcriptional elements the *MORp* construct will need to co-opt to drive transcription.
