# Supplementary material for: Human OPRM1 and murine Oprm1 promoter driven viral constructs for genetic access to μ-opioidergic cell types
Source: Nat Commun. 2023 Sep 13;14:5632. doi: 10.1038/s41467-023-41407-2 (PMC10499891; doi:10.1038/s41467-023-41407-2)
Supplement: Supplementary file 3 — Reporting Summary [file 41467_2023_41407_MOESM3_ESM.pdf]

nature portfolio

Corresponding author(s): Dr. Gregory Corder
Dr. Karl Deisseroth

Last updated by author(s): Jul 19, 2023

Reporting Summary

Nature Portfolio wishes to improve the reproducibility of the work that we publish. This form provides structure for consistency and transparency in reporting. For further information on Nature Portfolio policies, see our Editorial Policies and the Editorial Policy Checklist.

Statistics

For all statistical analyses, confirm that the following items are present in the figure legend, table legend, main text, or Methods section.

n/a	Confirmed	
☐	☒	The exact sample size (*n*) for each experimental group/condition, given as a discrete number and unit of measurement
☐	☒	A statement on whether measurements were taken from distinct samples or whether the same sample was measured repeatedly
☐	☒	The statistical test(s) used AND whether they are one- or two-sided<br>*Only common tests should be described solely by name; describe more complex techniques in the Methods section.*
☐	☒	A description of all covariates tested
☐	☒	A description of any assumptions or corrections, such as tests of normality and adjustment for multiple comparisons
☐	☒	A full description of the statistical parameters including central tendency (e.g. means) or other basic estimates (e.g. regression coefficient) AND variation (e.g. standard deviation) or associated estimates of uncertainty (e.g. confidence intervals)
☐	☒	For null hypothesis testing, the test statistic (e.g. *F*, *t*, *r*) with confidence intervals, effect sizes, degrees of freedom and *P* value noted<br>*Give P values as exact values whenever suitable.*
☒	☐	For Bayesian analysis, information on the choice of priors and Markov chain Monte Carlo settings
☒	☐	For hierarchical and complex designs, identification of the appropriate level for tests and full reporting of outcomes
☒	☐	Estimates of effect sizes (e.g. Cohen's *d*, Pearson's *r*), indicating how they were calculated

Our web collection on statistics for biologists contains articles on many of the points above.

Software and code

Policy information about availability of computer code

Data collection	Imaging data was collected using both Keyence BZ-X Analyzer and Viewer software (v. 1.4.0.1) and Zeiss Zen microscopy software (v. 3.4). In vivo fiber photometry data was collected using the Synapse software suite (v. 96) from Tucker-Davis Technologies. No specialized software was used for the collection of any reported behavioral data. Select behavioral data was analyzed via the use of the freeware analysis tool BORIS (v. 8.20).
Data analysis	All statistical analyses were performed using Graphpad Prism 9 & 10 software. Image data analyses were performed using both Fiji/ImageJ (v. 2) and Adobe Photoshop (v. 23.5) software suites. In vivo fiber photometry data was analyzed using both the MATLAB (v. 9.11) software package pMAT (v. 1-2) and the electrophysiological software suite pClamp (v. 10.6) from Molecular Devices.

For manuscripts utilizing custom algorithms or software that are central to the research but not yet described in published literature, software must be made available to editors and reviewers. We strongly encourage code deposition in a community repository (e.g. GitHub). See the Nature Portfolio guidelines for submitting code & software for further information.

Data

Policy information about availability of data

All manuscripts must include a data availability statement. This statement should provide the following information, where applicable:

- Accession codes, unique identifiers, or web links for publicly available datasets
- A description of any restrictions on data availability
- For clinical datasets or third party data, please ensure that the statement adheres to our policy

All source and raw data generated in this study have been deposited in the Zenodo database under the accession code (). Additional requests for further data or information regarding available data files will be addressed upon request to the corresponding authors.

Human research participants

Policy information about studies involving human research participants and Sex and Gender in Research.

Reporting on sex and gender	N/A
Population characteristics	N/A
Recruitment	N/A
Ethics oversight	N/A

Note that full information on the approval of the study protocol must also be provided in the manuscript.

Field-specific reporting

Please select the one below that is the best fit for your research. If you are not sure, read the appropriate sections before making your selection.

☒ Life sciences ☐ Behavioural & social sciences ☐ Ecological, evolutionary & environmental sciences

For a reference copy of the document with all sections, see nature.com/documents/nr-reporting-summary-flat.pdf

Life sciences study design

All studies must disclose on these points even when the disclosure is negative.

Sample size	No statistics were used to determine sample sizes. Sample sizes for chemogenetic and fiber photometry testing were chosen based on common practices that have historically been reported within the murine behavioral research field, including instances from previously peer reviewed publications authored by the lead and communicating authors (see PMID: 30655440, PMID: 32277042, PMID: 32074627 and PMID: 36941364 for examples). Standard practices reported for average sample sizes deemed sufficient to allow for well powered statistical analyses of both in situ and immunohistochemical studies for examining transcript or protein expression profiles across animals were also applied as previously reported in the literature (see PMID: 25600267 and PMID: 32277042 for previous, peer-reviewed examples). Sample sizes were not taken into account for experiments in which the end goal was qualitative, not quantitative, and primarily based on the ability to determine successful and efficient transduction of neural tissue via one of our viruses. In these instances, as reported in the manuscript text and additional reporting documents, cohort sizes for each set of injections with these viruses were set at a high enough number to hopefully allow for the successful transduction of target sites in at least one animal (groups typically consisted of 5+ animals per virus and per set of injections). As previous work in our lab in done to test or injection animals with new or untested virus had shown that at least 2-3 animals would be sufficient to account for any technical error that could occur over the course of a single stereotaxic surgery, a slight larger number of animals for this testing was deemed sufficient.
Data exclusions	For all imaging and behavioral studies, virus injected animals with either little or no evidence of viral transduction and/or incorrect viral targeting were excluded from any final analyses. Poor or loss of coupling between the fiber optic patch cord and the fiber optic implant on mice during fiber photometry testing led to the data collected from that animal for a given test to be discarded. No other mice or data points were excluded across analyses.
Replication	Virus transduction efficiency, specificity and restriction patterns were reliably reproduced across multiple cohorts of animals from experimental species discussed in the text (where applicable). Experimental findings in imaging studies regarding viral specificity were found to be reliably replicated across multiple cohorts of animals. In behavioral testing, findings within experimental and control groups were also found to be readily replicated. As multiple cohorts were not used for such studies, steps were taken to ensure that group sizes for all behavioral studies were sufficiently powered, as per standard animal behavioral research practices. Indication of data from individual subjects across all tests are displayed in figures where applicable. Detailed information and protocol outlines are additionally provided in figure legends and the Methods section, respectively, to ensure reproducibility.
Randomization	For spinal chemogenetic behavioral experiments, the order of group testing was randomized, but not blinded, such that we alternated testing mMORp-hM4Di-mCherry and then hSyn-mCherry control mice to control for testing order and time of day. For in vivo fiber photometry

testing, group testing was not randomized, with experimental and control virus injected animal groups run consecutively over the course of the testing day, with the experimental group run first. Randomization and blinding were not deemed possible for these studies, as the simultaneous calcium event recording that was conducted during behavioral testing would have immediately identified GCaMP6f+ mice and eYFP+ control mice due to the presence of detectable signal for GCaMP activity in the experimental group mice and the complete lack of this signal in control group mice (as demonstrated by the relevant data and figures provided for these studies in our manuscript). As such, animals were run as separate experimental and control groups for these studies, with photometry and behavioral data scored after initial acquisition. Following this logic, chronic morphine drinking and saccharin drinking groups were run consecutively and as block groups as well during fiber photometry testing. For all other histological and viral validation based experiments discussed in this manuscript, randomization was also not applied, as it for the purposes of either of these sets of quantitative or qualitative analyses, random allocation across experimental groups was not deemed necessary. As the end goal of our quantitative histological studies was specifically to determine if our viral tools did indeed more selectively transduce MOR/Oprm1/OPRM1+ cells more selectively over other cell types within the same tissue sample, group allocation was not necessary (nor randomization needed) as all animals used in these studies were injected with the same experimental viruses. Similarly, for viral validation studies, as the end results of these experiments was deemed to be observation of either successful viral transduction or not, knowledge of the injected viruses was already known, and confirmation of expression (or lack there was) was deemed to be a fairly definitive outcome, and not amenable (or necessary) to be subject to randomization.

Blinding	For chemogenetic behavioral testing, data were analyzed by a second experimenter, blinded to group identification. Order and identity of experimental or control virus injected groups were not blinded for initial in vivo fiber photometry testings or data analysis as the identity of these groups was given away with the presence of measurable calcium mediated events for initial testing. These initial tests also served primarily to explore the functionality of this viral tool more than anything as well. Built upon this same basis, experimenters were not blinded to group identification for acute morphine treatment or chronic morphine drinking assays. For all other histological and viral validation testing designed to assess both viral specificity and overall transduction efficiency, blinding was deemed as not possible or unnecessary across groups as the end goal of these studies was either to quantify the overall transduction specificity of an already known viral species or to simply validate the successful or unsuccessful expression of a viral species at injections sites across different neural structures. Even in the case where different viruses were co-injected for select specificity assays, specific fluorophores associated with and necessary to identify which virus was able to transduce which cells, made the identity of each viral species used difficult to blind experimenters to, and somewhat unnecessary, as it was crucial to be able to distinguish one type of virus from another for the successful interpretation and quantification of the results for these studies in general. As no specific manipulations were performed on the animal cohorts that compromised each of the viral injection groups across all histological assays (both IHC and ISH based), no additional blinding was deemed necessary for the effective analysis of these experiments.

Reporting for specific materials, systems and methods

We require information from authors about some types of materials, experimental systems and methods used in many studies. Here, indicate whether each material, system or method listed is relevant to your study. If you are not sure if a list item applies to your research, read the appropriate section before selecting a response.

Materials & experimental systems

n/a	Involved in the study
☐	☒ Antibodies
☐	☒ Eukaryotic cell lines
☒	☐ Palaeontology and archaeology
☐	☒ Animals and other organisms
☒	☐ Clinical data
☒	☐ Dual use research of concern

Methods

n/a	Involved in the study
☒	☐ ChIP-seq
☒	☐ Flow cytometry
☒	☐ MRI-based neuroimaging

Antibodies

Antibodies used	Primary antibodies:<br>Mouse anti-NeuN (EMD Millipore, MAB377, clone A60)<br>Chicken anti-GFP (Abcam, ab13970)<br>Chicken anti-RFP (Novus, NBP1-97371)<br>Rabbit anti-Iba1 (Wako, 019-19741)<br>Rabbit anti-Cre (Synaptic Systems, 257 003)<br>Rabbit anti-MOR (Abcam, ab134054, clone UMB3)<br>Mouse anti-APC (Millipore, OP80, clone CC-1)<br>Goat anti-PDGFRa (R&D, AF1062)<br>Rabbit anti-GFAP (Agilent, Z033429-2)<br><br>Secondary Antibodies:<br>Alexa-Fluor 647 Donkey anti-mouse (Thermo Scientific, A31571)<br>Alexa-Fluor 647 donkey anti-rabbit (Thermo Scientific, A31573)<br>Alexa-Fluor 488 donkey anti-chicken (Jackson Immuno, 703-545-155)<br>Alexa-Fluor 555 donkey anti-rabbit (Thermo Scientific, A31572)<br>Alexa-Fluor 594 donkey anti-chicken (Jackson Immuno, 703-585-155)<br>Alexa-Fluor 594 donkey anti-goat (Thermo Scientific, A11058)

Validation	EMD Millipore antibodies are routinely evaluated by immunohistochemistry on brain tissue from multiple species of interest in order to validate their efficacy. Additional details for MAB377 can be found at: https://www.emdmillipore.com/US/en/product/Anti-NeuN-Antibody-clone-A60,MM_NF-MAB377#overview, and supporting citations for it's application in rodent brain tissue can be found at: https://www.emdmillipore.com/US/en/product/Anti-NeuN-Antibody-clone-A60,MM_NF-MAB377#documentation. Anti-APC (Ab-7), mouse monoclonal, clone CC-1, recognizes APC in oligodendrocytes and astrocytes. It is validated for ICC, IF, free-floating Sections, and paraffin and frozen sections. Additional information can be found at: https://www.emdmillipore.com/US/en/product/Anti-APC-Ab-7-Mouse-mAb-CC-1,EMD_BIO-OP80.<br><br>All Abcam antibodies have been tested and approved for use in immunohistochemistry for neural tissue across multiple species, with both validation specs and supporting references for their use in mammalian tissue available at: https://www.abcam.com/gfp-antibody-ab13970.html (for anti-GFP), and https://www.abcam.com/mu-opioid-receptor-antibody-umb3-c-terminal-ab134054.html (for anti-MOR).<br><br>Novus antibody NBP1-97371 has been validated for use in immunohistochemistry in multiple tissue types, including neural tissue from rodents, with additional supporting material and documentation available at: https://www.novusbio.com/products/rfp-antibody_nbp1-97371#datasheet.<br><br>Wako antibody 019-19741 has been validated for reactivity in neural tissue and for use in immunohistochemistry across multiple tissues types and in numerous publications (3,000+). Additional information can be found at: https://labchem-wako.fujifilm.com/us/product/detail/W01W0101-1974.html.<br><br>R&D Systems antibody AF1062 detects mouse PDGF R alpha in direct ELISAs and Western blots. In direct ELISAs, less than 1% cross-reactivity with recombinant human (rh) PDGF R alpha, rhPDGF R beta, and recombinant mouse PDGF R beta is observed. These results have been validated across multiple tissue and lysate types, and presented across several publications. Additional information can be found at: https://www.rndsystems.com/products/mouse-pdgf-ralpha-antibody_af1062?gclid=CjwKCAjwvJyjBhApEiwAWz2nLREAMkxkaqvivHX5HMFr7eN4W8hEc34YgmhavDvSezk4eiPp7tpbqxoCzDYQAvD_BwE&gclsrc=aw.ds.<br><br>Aligent antibody Z033429-2 for GFAP has been tested and approved for use use in immunohistochemical studies (Glial Fibrillary Acidic Protein, Concentrate. Polyclonal Rabbit Anti-, Concentrated Antibody for Manual Use, Unconjugated, Immunohistochemistry, Ig fraction, Immunohistochemistry, 1 mL). Additional information can be found: https://www.agilent.com/store/en_US/Prod-Z033429-2/Z033429-2.<br><br>Synaptic Systems antibody 257 003 for Cre recombinase has been tested and approved for use in immunohistochemistry and for specificity in neural tissue across several recent publications. These and additional details can be found at: https://sysy.com/product/257003.

Eukaryotic cell lines

Policy information about cell lines and Sex and Gender in Research

Cell line source(s)	LiPSC-GR1.1 cells (Lonza) served as the base cell iPS (single donor, host sex: male, source: umbilical cord).<br>Human astroglial line A172 (ATCC CRL-1620), human microglial C20 cells, human neuronal SH-SY5Y cells (American Type Culture Collection, ATCC CRL-2266) and murine N9 microglial cells.
Authentication	LiPSC-GR1.1: line authentication assays performed as reported in PMID: 27283945 & PMID: 26411904.<br>A172: line authentication reported by manufacturer (https://www.atcc.org/products/crl-1620) .<br>C20: line authentication assays performed as reported in PMID: 27873219.<br>SH-SY5Y: line authentication reported by manufacturer (https://www.atcc.org/products/crl-2266).<br>N9: line authentication assays performed as reported in PMID: 18701698, PMID: 2789141 & PMID: 7681038.
Mycoplasma contamination	LiPSC-GR1.1: negative for mycoplasma (testing reported in PMID: 27283945)<br>A172: negative for mycoplasma (see manufacturer's website for reporting: https://www.atcc.org/products/crl-1620)<br>C20: reported negative by individual labs; line not maintained by a manufacturer.<br>SH-SY5Y: negative for mycoplasma (see manufacturer's website for reporting: https://www.atcc.org/products/crl-2266)<br>N9: reported negative by individual labs; additionally reported negative by separate manufacturers (see https://neuros.creative-biolabs.com/mouse-microglia-n9-44803.htm).
Commonly misidentified lines<br>(See ICLAC register)	No commonly misidentified cell lines were used in studies conducted for this manuscript.

Animals and other research organisms

Policy information about studies involving animals; ARRIVE guidelines recommended for reporting animal research, and Sex and Gender in Research

Laboratory animals	Mice:<br>Male, wildtype C57BL/6J mice (Jackson Laboratories, 000664); ages 8 - 20 wks<br>Male, heterozygous Sst-IRES-Cre mice (Jackson Laboratories, 013044; provided by Dr. Marc Fuccillo, UPenn); ages 8 - 10 wks<br>Male and female, heterozygous Oprm1-Cre:GFP mice (Jackson Laboratories, 035574); ages 8 - 12 wks<br>Male and female, heterozygous and homozygous Oprm1-2A-Cre:Sun1-sfGFP mice (provided by the lab of Dr. Julie Blendy, UPenn);

	ages 8 -12 wks<br><br>Rats:<br>Male, wildtype Sprague-Dawley rats (Charles River, 400; provided by the lab of Dr. Matthew Hayes, UPenn); ages 10 - 20 wks<br><br>Shrews:<br>Female, wildtype Asian house shrews (provided by the lab of Dr. Matthew Hayes, UPenn); aged 10 - 20 wks<br><br>Rhesus Macaque:<br>Male, rhesus macaque; aged five years
Wild animals	This study did not use any wild animals.
Reporting on sex	This study utilized both male and female mice (specified above), male rats, female shrews, and a single male rhesus macaque.
Field-collected samples	This study did not involved any samples collected from the field.
Ethics oversight	All procedures and manipulations conducted in all animal species were approved by the Institutional Animal Use and Care Committees of the University of Pennsylvania, Stanford University and Brigham and Women's Hospital, Harvard Medical School.

Note that full information on the approval of the study protocol must also be provided in the manuscript.
